# The vulnerability of radical SAM enzymes to oxidants and soft metals

**DOI:** 10.1016/j.redox.2022.102495

**Published:** 2022-10-07

**Authors:** Sanjay Kumar Rohaun, James A. Imlay

**Affiliations:** Department of Microbiology, University of Illinois, Urbana, IL, 61801, USA

**Keywords:** Reactive oxygen species, Copper, Nitric oxide, Iron-sulfur clusters

## Abstract

Radical S-adenosylmethionine enzymes (RSEs) drive diverse biological processes by catalyzing chemically difficult reactions. Each of these enzymes uses a solvent-exposed [4Fe–4S] cluster to coordinate and cleave its SAM co-reactant. This cluster is destroyed during oxic handling, forcing investigators to work with these enzymes under anoxic conditions. Analogous substrate-binding [4Fe–4S] clusters in dehydratases are similarly sensitive to oxygen in vitro; they are also extremely vulnerable to reactive oxygen species (ROS) in vitro and in vivo. These observations suggested that ROS might similarly poison RSEs. This conjecture received apparent support by the observation that when *E. coli* experiences hydrogen peroxide stress, it induces a cluster-free isozyme of the RSE HemN. In the present study, surprisingly, the purified RSEs viperin and HemN proved quite resistant to peroxide and superoxide in vitro. Furthermore, pathways that require RSEs remained active inside *E. coli* cells that were acutely stressed by hydrogen peroxide and superoxide. Viperin, but not HemN, was gradually poisoned by molecular oxygen in vitro, forming an apparent [3Fe–4S]^+^ form that was readily reactivated. The modest rate of damage, and the known ability of cells to repair [3Fe–4S]^+^ clusters, suggest why these RSEs remain functional inside fully aerated organisms. In contrast, copper(I) damaged HemN and viperin in vitro as readily as it did fumarase, a known target of copper toxicity inside *E. coli*. Excess intracellular copper also impaired RSE-dependent biosynthetic processes. These data indicate that RSEs may be targets of copper stress but not of reactive oxygen species.

## 1 Introduction

Radical S-adenosylmethionine enzymes (RSEs) use radical-based mechanisms to catalyze a wealth of difficult chemistry [1,2]. These enzymes underpin processes that are especially common in microbes. While humans express eight RSEs, *E. coli* possesses at least twenty two (Table S1), which contribute to the activation of glycyl-radical enzymes, the modification of specialized RNAs, and the synthesis of cofactors (https://biocyc.org). Even more RSEs are found in microbes that synthesize secondary metabolites.

**Fig. 1:**
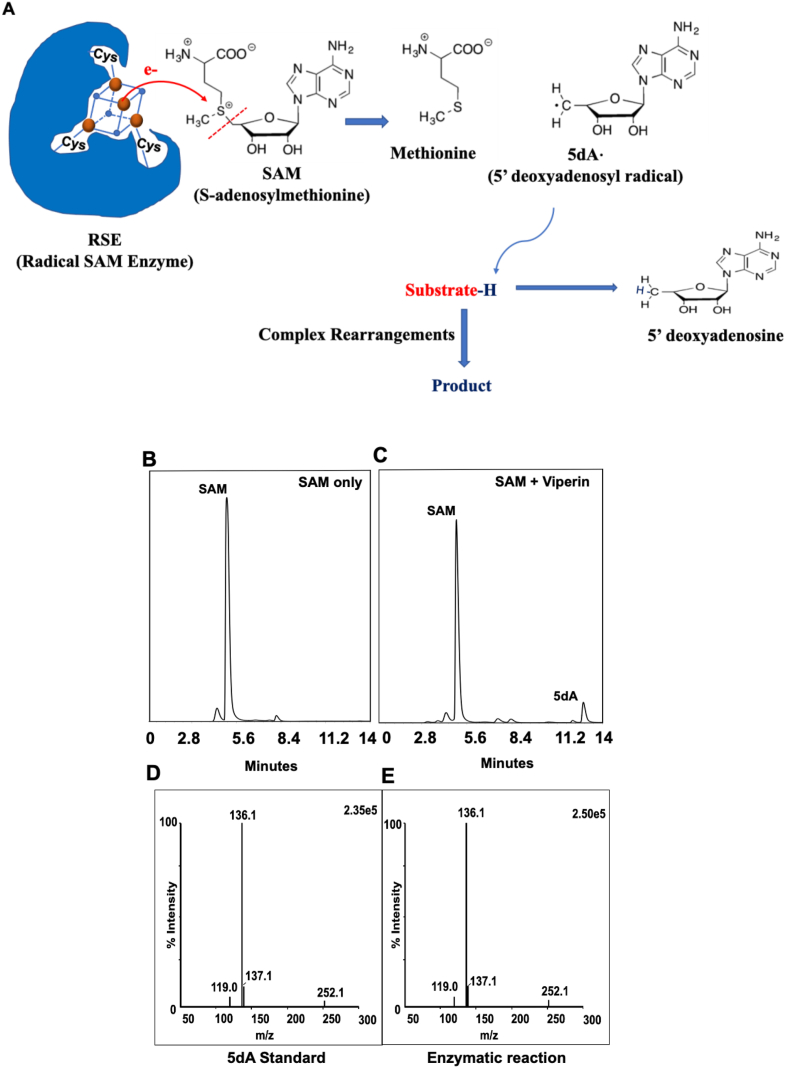
SAM cleavage activity of radical SAM enzymes (RSEs). (A) Schematic for reactions catalyzed by RSEs. RSE (blue) with a [4Fe–4S] cluster (maroon and blue) coordinates a S-adenosylmethionine (SAM) molecule. The [4Fe–4S]^+^ species then transfers an electron to SAM, splitting it into methionine and an adenosyl radical (Ad^.^). The Ad^.^ radical abstracts an electron from the substrate, initiating catalysis. (B–C) HPLC analysis of SAM cleavage by viperin. (B) SAM elutes from a C_18_ column with a major peak at 5.5 min (at 254 nm). (C) Viperin with an intact [4Fe–4S] cluster cleaves SAM reductively, leading to the formation of 5 dA^**⋅**^ radical. In the absence of substrate, the 5 dA^**⋅**^ radical is reduced by dithionite to form 5 dA, which elutes at 12.7 min (See Materials and Methods for details). (D) LC-MSMS of a 5 dA standard. (E) LC-MSMS of the 5 dA fraction generated by the enzymatic reaction of viperin and SAM. (For interpretation of the references to colour in this figure legend, the reader is referred to the Web version of this article.)

A universal feature of RSEs is a [4Fe–4S]^2+^ cluster that coordinates S-adenosylmethionine (SAM) via its carboxylate and amine moieties. Reactions initiate with the binding of enzyme substrate and the transfer of a single electron from a low-potential donor, likely flavodoxin, to this cluster. The [4Fe–4S]^+^ species then pushes the electron to SAM, splitting it into methionine and an adenosyl radical (Ad^**.**^). The radical abstracts an electron from the enzyme substrate, and a variety of catalytic pathways follow (Fig. 1A).

To bind SAM the RSE [4Fe–4S] cluster must be at least momentarily exposed to solvent—a feature that is shared by the [4Fe–4S] clusters of aconitase-class dehydratases [3]. An unfortunate consequence of cluster exposure in dehydratases is their vulnerability to oxidants and soft metals, which are too small to be excluded from their active sites. Notably, superoxide and hydrogen peroxide, which are accidental by-products of metabolism, bind directly to the [4Fe–4S]^2+^ dehydratase clusters and univalently oxidize them to the [4Fe–4S]^3+^ valence [[4], [5], [6]]. This form is unstable, and it spontaneously releases iron, leaving behind a [3Fe–4S]^+^ cluster that is catalytically inactive. Damaged clusters can be reactivated by Fe(II)/DTT treatment in vitro, and they are reassembled in vivo with a halftime of ∼5 min [7,8]. Thus, the steady-state activities of these enzymes inside cells reflect the dynamic equilibrium between damage and repair. Because virtually all organisms possess high titers of superoxide dismutases, catalases, and peroxidases, the level of endogenous reactive oxygen species (ROS) is low, and dehydratase-dependent pathways remain functional inside aerobic cells [4]. In mutants that lack these scavenging enzymes, however, ROS levels rise, the enzymes exhibit little activity, and the biosynthetic and catabolic pathways that depend upon them are inactive [8].

Even in scavenger-proficient wild-type cells, dehydratases are threatened when cells encounter external sources of H_2_O_2_, which easily crosses membranes, or redox-cycling antibiotics, which amplify intracellular ROS production [9]. Hydrogen peroxide is produced by lactic acid bacteria, by chemical and photochemical processes in natural waters, and by the oxidative burst of the host immune system [10]. Redox-cycling compounds are excreted by a variety of microbes in an effort to kill their competitors. To cope with these stresses, *E. coli* and most microbes employ inducible defense regulons. The OxyR transcription factor of *E. coli* detects incoming H_2_O_2_ through the oxidation of its sensory cysteine residue, and it then activates the expression of a dozen defensive operons [[11], [12], [13]]. These include genes that encode catalase and peroxidase. Other induced proteins speed the repair of damaged clusters, import manganese to remetallate mononuclear Fe(II) enzymes, and sequester iron to limit DNA damage [14].

Because RSEs also possess solute-binding [4Fe–4S] clusters, it seems plausible that, like dehydratases, they will be highly sensitive to ROS. Several observations hint at this possibility. First, RSEs lose their clusters if they are purified or handled under oxic conditions [1,[15], [16], [17]]—a feature that is also observed for most [4Fe–4S] dehydratases. It has not been clear whether molecular oxygen itself, or ROS derived from it, are responsible for this damage. However, the fact that both dehydratases and RSEs are functional inside aerobic cells could suggest that the key oxidants are ROS, which scavenger enzymes keep at low levels in vivo.

Second, two groups have reported that copper, which displaces iron from dehydratase iron-sulfur clusters [18], can also impair heme biosynthesis in bacteria [[19], [20], [21]]. That pathway depends upon HemN, an RSE. If the bottleneck is indeed due to HemN damage, then this phenotype would appear to confirm that RSE clusters are exposed and vulnerable to small molecules.

Finally, we observed that *E. coli hemF*, which encodes a cluster-free, manganese-dependent isozyme of HemN, is induced by the OxyR regulon [22]. A reasonable explanation might be that H_2_O_2_ directly attacks the HemN cluster, so that the cell resorts to a non-RSE enzyme. Heme synthesis is especially critical during H_2_O_2_ stress, because the cell accelerates its synthesis of catalase by more than an order of magnitude [13].

In this report we show that sampled RSE enzymes are, contrary to expectation, unaffected by either H_2_O_2_ or superoxide (O_2_^−^). They exhibit some sensitivity to molecular oxygen itself, providing a rare example in which iron-sulfur clusters are damaged by oxygen but not by ROS. Still, the rate of damage by oxygen is modest and apparently insufficient to outstrip repair processes. This would explain why these enzymes can function inside air-saturated cells. We propose that H_2_O_2_-stressed *E. coli* replaces HemN with cluster-free HemF because these cells contain little iron for cluster building. Finally, we confirm that copper can poison RSEs and pathways that depend upon them.

## 2 Materials & methods

### 2.1 Reagents

Ammonium acetate, acetonitrile, phenylalanine, glycerol, and d-glucose were purchased from Fisher Scientific. Arabinose, 5-aminolevulinic acid (5-ALA), ascorbic acid, biotin, bovine liver catalase, chloramphenicol, citraconic acid, copper (II) sulfate, 5′deoxyadenosine (5 dA), 5′-deoxy-5’-(methylthio)adenosine (MTA), diethylamine NONOate, diethylenetriamine pentaacetic acid (DTPA), dithiothreitol (DTT), ferrous ammonium sulfate (FAS), horseradish peroxidase, hydrogen peroxide, imidazole, isopropyl β-d-1-thiogalactopyranoside (IPTG), lipoic acid, malic acid, potassium ferricyanide, S-adenosylmethionine (SAM), silver nitrate, superoxide dismutase (SOD), sodium dithionite**,** xanthine, xanthine oxidase, histidine, cystine, isoleucine, methionine, tyrosine, tryptophan, and valine were obtained from Sigma. Amicon centrifugal filters were from Millipore, and Amplex Red was from Invitrogen. His GraviTrap™ was purchased from GE Healthcare.

### 2.2 Bacterial strains

The bacterial strains used in this study are listed in Table 1. Gene deletions were generated by the lambda-Red recombinase method [23]. Deletions were verified by PCR. P1 transduction [24] was used to move mutations between strains. All genetic manipulations in the SOD^−^ or Hpx^−^ backgrounds were performed in the anaerobic chamber to avoid any enrichment of mutants bearing suppressor mutations.

**Table 1:** List of strains used in the study.

Strain	Genotype	Source or Reference
MG1655	F^−^ wild-type	*E. coli* Genetic Stock Center
BW25113	F^−^*lacI rrnB* Δ*lacZ hsdK* Δ*araBAD* Δ*rhaBAD*	Lab collection
W3110	F^−^ wild-type	Lab collection
LC106	MG1655 Δ*ahpF*::*kan* Δ(*katG17*::Tn*10*)*1* Δ(*katE12*::Tn*10*)*1*	[87]
SR026	As LC106 plus Δ*thiC1*::*cat*	This study
SR028	As LC106 plus Δ*lipA1*::*cat*	This study
SR030	As LC106 plus Δ*bioB1*::*cat*	This study
SMA1489	As LC106 plus Δ*hemF1*::*cat*	Lab collection
KI232	As MG1655 plus (Δ*sodA*)*1* (Δ*sodB::kan*)*1-*Δ*2*	Lab collection
SR032	As KI232 plus Δ*thiC1::cat*	This study
SR034	As KI232 plus Δ*lipA1::cat*	This study
SR036	As KI232 plus Δ*bioB1::cat*	This study
SR038	As KI232 plus Δ*hemF1*::*cat*	This study
SMA1140	As MG1655 plus Δ*hemA1::cat*	Lab collection
LEM33	As W3110 plus Δ*copA::kan* Δ*cueO* Δ*cusCFBA*	[18]
BL21(DE3)	F^−^*ompT hsdS*_*B*_ (*r*_*B*_^−^*m*_*B*_^−^) *gal dcm* (λ DE3)	Lab collection
SR011	As BL21(DE3) plus *katG*Δ*FG ∼ zij-8::cat* Δ*katE*::Tn*10*	This study
SMA1367	As BW25113 plus *katG*Δ*FG ∼ zij-8::cat*	Lab collection
JI360	As MG1655 plus *katE12*::*Tn10*	Lab collection
SR021	As SR011 plus pSR010	This study
SR022	As SR021 plus pSR003	This study
SR040	As SR011 plus pLZ47	This study
SR048	As SR021 plus pSR012	This study
Plasmids used		
pSR003	pDuet plus a 834 bp fragment encoding *Methanofollis liminatans* viperin (with BamHI and EcoRI)	[26]
pSR010	pDB1282 with *IscSUA*-*hscAB*-*fdx* operon from *Azotobacter vinelandii*	[25]
pSR012	pET15b plus a 1374 bp fragment encoding *E. coli hemN* with NdeI and BamHI	This study
pLZ47	pET16b plus *E. coli fumA*	[27]

### 2.3 Gene cloning and protein purification

A catalase-deficient Δ*katG* Δ*katE* derivative of the protein-expression strain BL21(DE3) was generated by P1 transduction from lab strains (Table 1). This manipulation ensured that trace catalase could not contaminate purified proteins. The pDB1282-ISC plasmid, which overexpresses the *iscSUA-hscAB-fdx* operon of *Azotobacter vinelandii* [25], was transformed into the strain with kanamycin selection. The ISC system assembles iron-sulfur clusters and transfers them to client proteins; its overproduction boosts the activation of cluster-requiring enzymes.

The *hemN* gene (encoding coproporphyrinogen III dehydrogenase) was PCR amplified (see details of primers in Table 2) from the genomic DNA of the *E. coli* K-12 wild-type strain MG1655. The PCR product was inserted into the pET15b plasmid (double digested with NdeI and BamHI), and the construct was confirmed by sequencing. The viperin gene from *Methanofollis liminatans*, cloned in a pETDuet plasmid, was a gift of Dr. Raven Huang [26]. The *fumA* gene of *E. coli*, encoding a [4Fe–4S]-dependent fumarase, was inserted in a pET16b plasmid [27]. These ampicillin-selected constructs each express the cloned gene under the control of an IPTG-inducible T7 promoter system.

**Table 2:** List of primers used in the study.

*hemN* cloning	5′ ATATACATATGTCTGTACAGCAAATC 3′
	5′ TATATGGATCCAATCACCCGAGAGAACTG 3′
*bioB* deletion	5′ GGCTCACCGCCCACGCTGGACATTGTCGCAAGTCACTGTAG GCTGGAGCTGCTTCG 3′
	5′ ATAATGCTGCCGCGTTGTAATATTCGTCGGTGTCCGCATATGA ATATCCTCCTTAG 3′
*lipA* deletion	5′ AGTAAACCCATTGTGATGGAACGCGGTGTTAAATACTGTAGG CTGGAGCTGCTTCG 3′
	5′ CTTAACTTCCATCCCTTTCGCCTGCAAATCGGCGTCATATGA ATATCCTCCTTAG 3′
*thiC* deletion	5′ GTCTGCAACAAAACTGACCCGCCGCGAACAACGCGCTGTAGG CTGGAGCTGCTTCG 3′
	5′ ACGCTTCCTCCTTACGCAGGTAGATTTCTCCGCCTCCATATG AATATCCTCCTTAG 3′
Primers for *trxC* qRT PCR	5′ GACTTGTTTGACGGAGAGGTGATTA 3′
	5′ TGCCCAGAAGTCGATCACCAC 3′
Primers for *gmk* qRT PCR	5′ ATATTGTTTCTGCCCCCGT 3′
	5′ GTCGCCAGTACTTGCTCAAT 3′

Cells were grown in 37 °C LB medium in hypoxic conditions by cultivating a 1.2-liter culture in a 2-liter flask with shaking at 220 rpm. When the culture reached an OD_600_ = 0.4, l-arabinose (0.2% final) was added to induce the ISC operon. At OD_600_ = 0.8, cells were shifted to 18 °C, and after 15 min IPTG (100 μM) was added to induce synthesis of the RSE. Ferrous ammonium sulfate (50 μM) was also added. Cultures were incubated at 18 °C for 16 h with shaking at 120 rpm. Cells were then centrifuged and washed with ice-cold buffer (20 mM Tris, pH 7.5 and 100 mM NaCl) before lysis. Storing cells at this stage at −80 °C (for 1–2 months) did not change protein yield or activity. For lysis, 4 g of cell pellet was resuspended in the anaerobic chamber in 25 ml of ice-cold anoxic buffer A (20 mM Tris, pH 7.5, 100 mM NaCl, and 5% glycerol). The resuspended cells in anaerobic buffer were quickly passed one time through a French press at 4 °C aerobically, and the lysate was shifted immediately back to the anaerobic chamber. The lysate was mixed by pipetting (to facilitate gas exchange), and it was then aliquoted into 2 ml Eppendorf tubes and centrifuged at 13000 rpm for 40 min at 4 °C. The supernatant was filtered using a 0.45 μm syringe filter in the anaerobic chamber, and it was then loaded onto a Ni-Sepharose™ column (GE) that had been stored anoxically and pre-equilibrated with ten column volumes of anoxic buffer A. The supernatant was loaded, the column was washed with with 10 column volumes of ice-cold buffer A containing 5 mM imidazole/250 mM NaCl, pH 7.5. The second wash involved 10 column volumes of 10 mM imidazole in buffer A. The third wash was with 5 column volumes of buffer A containing 20 mM imidazole. Protein was then eluted with 120 mM imidazole in buffer A; the brown fraction containing the iron-sulfur protein was collected. The eluted protein in the elution buffer was only stable for one day at 4 °C and lost activity if stored at −80 °C. However, the protein was stabilized by increasing the glycerol concentration to 10%. The protein fraction with 10% glycerol was frozen in the anaerobic chamber using dry ice and finally stored at −80 °C aerobically at a concentration of 300–500 μM. Under this condition the protein was stable for months and active for SAM cleavage assay in our assay system; however, the results presented here were obtained in experiments conducted on the same day of purification.

Imidazole was removed only to test whether it had any effect on protecting the enzyme against different oxidants. To remove imidazole, 200 μl eluted protein (500 μM) was diluted into cold anoxic buffer B (14.8 ml of 20 mM Tris, pH 7.5, 100 mM NaCl, 10% glycerol) and transferred to an Amicon® ultra-15 10K centrifugal filter at 4 °C in the anaerobic chamber. The column was spun at 5000 × *g* for 60 min at 4 °C to obtain a 200 μl sample. This step was performed three times.

Protein concentration was determined by UV. Protein extinction coefficients at 280 nm—50310 M^−1^ cm^−1^ for His-tagged HemN, 33920 M^−1^ cm^−1^ for His-viperin, and 55240 M^−1^ cm^−1^ for His-fumarase—were calculated using the Expasy website (https://web.expasy.org/protparam/). Note that to make solutions anoxic, buffers and ddH_2_O were autoclaved and transferred into anaerobic chamber while hot. The solutions were then kept in the anaerobic chamber for at least 3 days to make them fully anoxic before use in experiments.

The cysteine desulfurase IscS was purified and assayed as described [28].

### 2.4 Iron measurement

Iron content was determined by a Ferene-S assay [29]. Five μM protein in 200 μl sodium acetate buffer (50 mM, pH 5.5) was treated with 30 μl 12 N HCl and incubated at 25 °C for 10 min with shaking. Then 25 μl of 80% TCA was added, and the solution was incubated at 4 °C for 10 min. The sample was spun at 10,000 × *g* for 10 min. To 225 μl supernatant, 50 μl of acetic acid (45%) and 725 μl of Ferene-S solution (0.63 mM Ferene-S and 8.5 mM l-ascorbic acid in 3 M sodium acetate, pH 5.5) were added, and the mix was incubated at 25 °C for 10 min. Absorbance was measured at 595 nm. Standard solutions of FeCl_3_ were prepared in a similar way. Note that FeCl_3_ is soluble only at higher concentrations; therefore, a stock of 50 mM was prepared in ddH_2_O prior to dilution into the assay mixture.

### 2.5 Growth curves

Minimal glucose medium was prepared with minimal A salts, 0.2% glucose, and 5 mM MgSO_4_ [24]. For experiments that tested the ability of Hpx^−^ or SOD^−^ mutants to synthesize enzyme cofactors, the medium was supplemented with 0.5 mM of l-cystine, l-isoleucine, l-leucine, l-phenylalanine, l-methionine, l-valine, l-tyrosine, and l-tryptophan. Hpx^−^ mutants (see Table 1 for details) lack the catalases and peroxidases that scavenge endogenous hydrogen peroxide, and SOD^−^ mutants lack the manganese- and iron-cofactored superoxide dismutases. Consequently, endogenous ROS poison the branched-chain, aromatic, and sulfurous amino acid synthesis pathways in these cells, which therefore require amino-acid supplements to grow [4,30,31]. For thiamine experiments, we found that these amino acids had some contaminating thiamine, and so a lower concentration of 0.005 mM was added into the growth medium. Anaerobic cultures were grown in a Coy chamber (Coy Laboratory Products) with a gas phase of 90% N_2_, 5% H_2_, and 5% CO_2_. Medium was autoclaved and transferred into the anaerobic chamber while hot. It was stored in the anaerobic chamber for at least 3 days to make it fully anoxic before it was used in experiments.

Tests of cofactor synthesis required generating log-phase cultures in medium containing vitamins, removing the vitamins, and then monitoring outgrowth. The growth tests accommodated the fact that *E. coli* can persevere for several generations after vitamins are removed. Because some strains lacked scavenging systems, precultures were performed under anoxic conditions. Cells from glycerol stocks were streaked onto minimal glucose agar with required vitamins (4 nM biotin for *ΔbioB* mutants, 25 nM lipoate for *ΔlipA*, 5 nM thiamine for *ΔthiC*, and 5 μM 5-aminolevulinate for *ΔhemA*). Colonies were inoculated, and cultures were grown anaerobically overnight on the same media. The overnight cultures were then diluted to OD_600_ 0.005, and cells were grown to an OD_600_ of 0.1 in anaerobic chamber. Cells were centrifuged and washed thrice with a 10x volume of glucose medium devoid of vitamins. Cells were then diluted to OD_600_ 0.005 into aerobic medium containing 8 amino acids (above) and grown at 37 °C with shaking to an OD_600_ of 0.1. Cultures were repeatedly subcultured by dilution and outgrowth until growth ceased for the auxotrophs lacking vitamins.

Copper toxicity was examined using a *copA cueO cus* mutant lacking the known copper defensive systems (Table 1). An overnight culture was grown in glucose medium supplemented with Ile, Leu, and Val. These supplements are necessary because copper poisons enzymes in the branched-chain biosynthetic pathway [18]. The overnight culture was diluted to OD_600_ = 0.001 in the same medium, with or without CuSO_4_. Where indicated, vitamins were added individually or as a group (4 nM biotin, 25 nM lipoate, and 5 μM thiamine). Growth was monitored over time at 37 °C aerobically.

### 2.6 SAM cleavage enzyme assays

The SAM-cleavage activities of the HemN and viperin proteins were measured by a slight modification of previous methods [26,32,33]. Product formation was measured under anoxic conditions, using reagents and buffers from which oxygen had been removed. The enzyme was diluted to 30 μM in 83 μl reaction buffer (20 mM Tris, pH 7.5 and 50 mM NaCl). The HemN reaction was initiated by the step-wise addition of 2 μl SAM (final concentration 200 μM), 5 μl DTT (final concentration 5 mM), and 10 μl freshly prepared dithionite (final concentration 5 mM), making the total reaction volume equal to 100 μl. For viperin, final concentrations of 2 mM SAM and 50 mM DTT was used [32]. Reactions were executed at 37 °C in the anaerobic chamber, and they were stopped by the addition of 100 μl of ice-cold ddH_2_O.

Each sample was then shifted aerobically to a 3 kDa Amicon centrifugal filter (0.5 ml; Millipore) and spun at 9000 × *g* for 15 min. The supernatant was collected and loaded onto an HPLC. The supernatant was subjected to reverse phase Waters 2695 HPLC system by injection onto a Luna® 5 μm C18(2) 100 Å, 250 × 2 mm column (Phenomenex). The column was equilibrated with 95% solvent A (50 mM ammonium acetate, pH 5.5) and 5% solvent B (100% acetonitrile). The following gradient was applied with solvent B: 0–6 min, 20% B; 6–14 min, 20% B; 14–15 min, 5% B. Standard samples of S-adenosylmethionine (SAM; 1 mM), methylthioadenosine (MTA; 0.5 mM), and 5′-deoxyadenosine (5′dAdo; 20 μM) were detected by absorbance at 254 nm. SAM eluted at 5.5 min, 5′dAdo at 12.7 min, and MTA at 15.5 min. The generation of 5 dA in the SAM cleavage assays by enzyme was confirmed by LCMSMS analysis.

### 2.7 LC-MSMS

The supernatant from SAM cleavage enzyme assays was injected directly onto a Waters Acquity UPLC BEH C18, 1.7 μm, 2.1 × 50 mm column connected to a Waters Synapt G2-Si high-definition mass spectrometer, and the reaction samples were analyzed in positive mode. Solvents used were (A) 10 mM ammonium acetate (pH 5.5) and (B) 40% acetonitrile and 60% H_2_O. A gradient of 0–50% B was applied.

### 2.8 Testing whether small-molecule stressors inactivate purified RSEs

Enzyme exposures to oxidants, small metals, and nitric oxide were performed in an anaerobic chamber at 27 °C (ambient chamber temperature) in a standard buffer (20 mM Tris, pH 7.5, and 50 mM NaCl). For peroxide inactivation experiments, 100 μM H_2_O_2_ was incubated with diluted enzyme (30 μM) in reaction buffer with or without DTPA (30 μM). The H_2_O_2_/enzyme incubation mix volume was 83 μl. At a set time point, damage was terminated and the assay started by adding SAM, DTT, and dithionite. Dithionite immediately scavenges H_2_O_2_. DTPA is a chelator that traps loose iron present (if any) in the solution and thereby suppresses non-specific peroxide-iron reactions. DTPA does not extract prosthetic metals from these metalloenzymes. We determined that the presence or absence of 30 μM DTPA had no impact upon enzyme damage, and so subsequent experiments were performed without DTPA.

Enzyme inactivation by molecular oxygen was tested in closed polypropylene PCR tubes inside the anaerobic chamber. The labelled volume of these tubes is 0.2 ml, but their actual total volume is 290 μl. Therefore, a 290 μl mixture was prepared that contained 30 μM enzyme (17.5 μl of 500 μM stock), 40.6 μl of anaerobic buffer, and 232.4 μl of air-saturated buffer, rendering a final concentration of 200 μM dissolved O_2_. This volume filled the tube to the top, and the tube was then capped to avoid gas exchange with the anaerobic chamber. In control tubes, either catalase (200 U/ml) or SOD (500 U/ml) or both were added. (Air-saturated buffer was transferred to the anaerobic chamber in screw capped vials and used once only.) At a selected time point, 83 μl of the mix was removed to another tube, and the oxygen exposure was ended and the assay started by the addition of SAM, DTT, and dithionite. Dithionite instantly reduces oxygen to water.

Superoxide exposure required superoxide formation by xanthine oxidase [34] in an aerobic buffer. The mixture was constructed in a similar way as for O_2_ exposure except that xanthine (14.5 μl of 1 mM stock) and 10 mU/ml xanthine oxidase (14.5 μl of 200 mU/ml) were added with or without SOD. In this situation, enzyme damage was driven by a combination of oxygen and superoxide.

Potassium ferricyanide was dissolved in H_2_O and added to a final concentration of 100 μM to inactivate 30 μM RSEs in anoxic Tris-NaCl buffer.

To test enzyme sensitivity to copper, a stock solution of Cu(I) was prepared by mixing 1 mM CuSO_4_ with 1 mM ascorbic acid in anaerobic reaction buffer. The reduction of Cu (II) to Cu(I) was immediate (30 s) and confirmed by the complete loss of the Cu (II) absorption peak at 630 nm. Histidine (10 μM) was added to help mobilize the copper during the enzyme-Cu(I) incubation [18]. Enzyme (30 μM) was incubated anaerobically with 100 μM Cu(I) at 27 °C (in 83 μl). After this incubation, the assay was then started by adding SAM, DTT, and dithionite. The DTT also blocked further enzyme damage by chelating copper.

For NO inhibition assay, 30 μM enzyme was exposed to 100 μM of DEA NONOate in anoxic reaction buffer. The assay was then started by adding SAM, DTT, and dithionate.

Fumarase A at 1 μM was exposed to the various stressors in the same Tris/NaCl reaction buffer as HemN and viperin. Fumarase activity was then assayed by dilution to 100 nM final concentration in a capped anaerobic cuvette containing anoxic 20 mM Tris, pH 7.5, and 50 mM l-malate as substrate (400 μl total volume). The reaction was monitored at 250 nm.

### 2.9 Reactivation of damaged enzymes

After 30 min exposure to O_2_, the 83 μl solution of viperin was reactivated by the addition of 5 mM dithiothreitol (1 μl of 0.5 M DTT), followed by 0.5 mM ferrous ammonium sulfate (1 μl of 50 mM stock) and 11 μl anoxic buffer. The mix (96 μl) was incubated in the anaerobic chamber at RT for 20 min. The enzyme assay was then started by the addition of 2 μl SAM and 10 μl dithionite. No reactivation occurred if either iron or DTT was omitted from the reactivation mix.

IscS is a cysteine desulfurase, and it has been used to rebuild [4Fe–4S] clusters that were degraded beyond the [3Fe–4S] stage [35]. In vitro reconstitution using IscS was performed by incubating 83 μl oxidized viperin with 0.5 mM ferrous ammonium sulfate (1 μl of 50 mM stock), 5 mM DTT (1 μl 0.5 M stock), 2.5 mM cysteine (1 μl 250 mM stock), and 0.07 μM purified IscS (10 μl) at RT for 2 h. The enzyme was then assayed by the addition of SAM and dithionite.

### 2.10 Gene expression analysis by qRT-PCR

Primary cultures of wild-type *E. coli* (MG1655) and its Hpx^−^ derivative were grown overnight at 37 °C anaerobically in glucose medium supplemented with eight amino acids (Phe, Try, Tyr, Leu, Ile, Val, Cys, and Met). Overnight cultures were diluted to OD_600_ 0.005 in anoxic glucose medium and grown at 37 °C to an OD_600_ of 0.1 without shaking in the anaerobic chamber. Cells were then moved out of the chamber and diluted to OD_600_ 0.005 in oxic glucose medium. These cells were grown with shaking at 37 °C to an OD_600_ of 0.4. The hot-phenol method was used for total RNA isolation, and RNA was collected from cells without centrifugation, to avoid the anoxia and H_2_O_2_ depletion that occurs inside a cell pellet. Briefly, 115 μl of 8x lysis buffer (3 M sodium acetate, 20% SDS, and 0.5 M EDTA; all RNAse free grade from Ambion) was added to 800 μl cell culture (without pelleting the cells) and vortexed to mix, followed by the addition of 915 μl hot (65 °C) phenol, pH 4.3 (Sigma). Samples were kept at 65 °C with shaking at 1400 rpm for 10 min. After centrifugation at 13,000 rpm for 10 min at RT, the top aqueous layer was removed and transferred to 700 μl of acid phenol-chloroform, pH 4.5 (Ambion). The sample was mixed by inversion and spun at 13,000 rpm for 10 min at RT. The top layer was removed, and the phenol-chloroform step was repeated twice. The top layer was removed and mixed into 1.3 ml of ethanol (100%) and kept at −80 °C overnight. The mix was spun at 12000 × *g* at 4 °C for 10 min. The pellet was washed with 500 μl 75% ethanol and spun again. The pellet was air-dried (15 min at RT) and resuspended in 20 μl TE buffer (RNAse free from Ambion). The isolated total RNA was treated with DNaseI (Qiagen). RNA was quantified using a Nanodrop spectrophotometer. cDNA was synthesized from 0.25 μg of RNA from each of the samples using a Superscript 1st strand cDNA Synthesis Kit (Invitrogen). For qRT-PCR analysis, 12.5 μl of the reaction was set, containing 6.25 μl of SYBR Green PCR Mix (BioRad), 2.5 μl of 1: 10 diluted template cDNA, 2.5 μl of 2 μM primer mix, and 1.25 μl of RNase-free water. The housekeeping Gmk gene encoding guanylate kinase was used as a reference internal control. The qRT-PCR was run at 95 °C for 30 s, followed by 39 cycles of denaturation at 95 °C for 5 s, and annealing and extension at 60 °C for 30 s. Amplification, data acquisition, and analysis were performed on an BioRad CFX Connect™ Real-time System. Data expressed as means ± standard errors were compared for statistical significance by unpaired *t*-test. The primer sequences used for qRT-PCR are listed in Table 2.

### 2.11 Measurements of hydrogen peroxide

The Amplex Red/horseradish peroxidase assay was used to measure H_2_O_2_ [36]. Aliquots for H_2_O_2_ measurements were removed at different time points from the 83 μl reaction mix containing 30 μM RSE enzyme and 100 μM H_2_O_2_ (in 20 mM Tris, pH 7.5, 50 mM NaCl, and 30 μM DTPA). A Shimadzu RF-150 was used to track fluorescence, with excitation at 520 nm and emission at 620 nm. For measurements of H_2_O_2_ in bacterial cultures, aerobic cultures were grown in glucose medium supplemented with eight amino acids to OD_600_ of 0.1. Aliquots were centrifuged at 13000 × *g*, and the supernatant was used for H_2_O_2_ measurements. Standard amounts of H_2_O_2_ were added to sterile medium in order to calibrate the system.

### 2.12 Isopropylmalate isomerase assay

Isopropylmalate isomerase (IPMI) activity was measured from cell lysates of aerobically grown cells (100 ml). Cells were cultured in our standard minimal glucose medium containing 8 amino acids. Overnight anaerobic cultures were inoculated into anaerobic medium (0.005 OD_600_), grown to 0.1 OD_600_, subcultured to 0.005 OD_600_ in aerobic medium, and grown to 0.1 OD_600_ with rigorous shaking. Cells were pelleted, shifted as a pellet to the anaerobic chamber, washed with 30 ml of anaerobic ice cold 100 mM Tris-Cl buffer (pH 7.5), and resuspended in anoxic 0.5 ml Tris-Cl buffer (100 mM, pH 7.5). Cells were lysed by sonication. Debris was removed by centrifugation (13K for 20 min at 4 °C in the chamber), and the supernatant was used for measurement of IPMI activity. The assay was constructed in a 0.5 ml capped cuvette that could then be moved to a spectrometer outside the chamber. Citraconic acid (0.4 mM) was employed as substrate at RT in the same Tris-Cl buffer. The decrease in absorbance at 235 nm was monitored, and specific activity was calculated (ΔA/min/mg).

### 2.13 Statistical analysis

All experiments were replicated at least in triplicate, and values reported as mean ± S.E.M. (standard error). In comparisons of multiple means, the results were analyzed by one way ANOVA (ANalysis Of VAriance) with post-hoc Tukey HSD (Honestly Significant Difference) test with a p < 0.01.

## 3 Results

Our inspection of RSEs focused upon HemN and viperin. These enzymes were selected due to their circumstantial connections to oxidative stress. When *E. coli* senses incoming H_2_O_2_, it induces HemF, a cluster-free isozyme of HemN, as if HemN itself were sensitive to H_2_O_2_ [37]. We sought to determine whether this is true. Viperin is an anti-viral protein found in all biological kingdoms. Microbes are likely to encounter phage bursts in oxidatively stressful environments, as H_2_O_2_ is a potent inducer of prophages [38,39]; therefore, viperin could help bystander microbes to fend off their own infection only if the RSE can remain active despite the presence of ROS. Similarly, mammalian viperin could only be effective if it can withstand the oxidative stress that arises from virus-associated inflammation. These logical arguments lead to opposite predictions for RSEs, and so both enzymes were examined.

We purified His-tagged HemN from *E. coli*. The *hemN* gene was cloned into a pET16b vector and overexpressed in a strain containing inactivating mutations in both genes encoding catalase. This feature ensured that the purified protein would not be contaminated by trace catalase, which otherwise might disrupt in vitro experiments. Overexpressed iron-sulfur proteins commonly exceed the capacity of cells to provide clusters to them, and so the Isc operon was co-expressed from another plasmid. (See Materials & Methods for details.) The enzyme was purified in an anaerobic chamber. The iron content of the purified protein indicated up to 55% occupancy with a [4Fe–4S] cluster (Table S2). The activity was not boosted by iron(II)/DTT, which typically reactivates oxidized [3Fe–4S] clusters; we therefore infer that the under-occupancy of the protein likely reflected insufficient de novo activation in vivo, rather than oxidative damage or simple iron dissociation in vitro. Further reactivation steps using purified IscS also proved unsuccessful, possibly signaling that the cluster-binding site in this apoprotein is poorly accessible to this treatment. Subsequent experiments utilized enzyme that had not undergone either of these reactivation procedures, with the benefit of avoiding ROS stress that can arise artifactually from free iron in the sample.

Like many RSEs, HemN initiates catalysis when its reduced [4Fe–4S] cluster cleaves SAM to methionine and a 5dA^**.**^ radical [33]. The 5dA^.^ radical then abstracts a hydrogen atom from substrate, forming 5-deoxyadenosine (5 dA) and triggering a subsequent rearrangement of the substrate. The cleavage of SAM is triggered by the binding of substrate, but even in the absence of substrate RSEs generate some 5dA^.^ radicals as a slow side reaction [40,41]. If this occurs under reducing conditions, methionine and 5 dA are released. Assays with native substrates are not always possible; for example, the HemN substrate is unstable to air and cannot be purchased or easily synthesized. Therefore, 5 dA production has often been used as a proxy for the [4Fe–4S] status of these enzymes [41]. We adapted the SAM cleavage assay from previous work with slight modifications [41]. After incubation of the HemN with SAM and the electron donor dithionite, reaction products were separated by HPLC and detected by UV absorbance. In the absence of enzyme, SAM samples presented a single peak at 5.5 min. The addition of enzyme created a peak that eluted at 12.7 min (Fig. 1C). This product was verified by LCMS to be 5 dA (Fig. 1E).

**Fig. 2:**
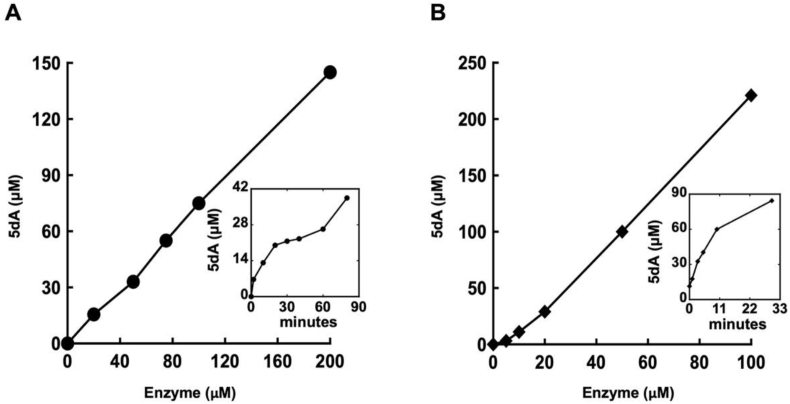
SAM cleavage by HemN and viperin. (A) Example of HemN enzyme activity. Anoxic reactions (20 min) contained 200 μM SAM, 5 mM DTT, 5 mM dithionite at 37 °C in anoxic 20 mM Tris, pH 7.5, and 50 mM NaCl. Inset: Enzyme activity (30 μM HemN) with varying time of reaction. (B) Viperin activity. Anoxic reactions (10 min) contained 2 mM SAM, 50 mM DTT, and 5 mM dithionite at 37 °C in the same anoxic buffer as HemN. Inset: Enzyme activity (30 μM enzyme) with varying time of reaction.

HemN SAM-cleavage activity was assessed in an anoxic reaction system containing 200 μM SAM, 5 mM DTT, and 5 mM dithionite for 20 min at 37 °C. Cold ddH_2_O was added to stop the reaction. The data showed progressive SAM cleavage as a function of enzyme amount or reaction time (Fig. 2A). Under these experimental conditions, 30 μM HemN and 200 μM SAM generated 12 μM 5 dA in 20 min (∼0.4 turnovers). This rate is comparable to that observed with other RSEs [32].

### 3.1 RSEs are not affected by H_2_O_2_ in vitro

**Fig. 3:**
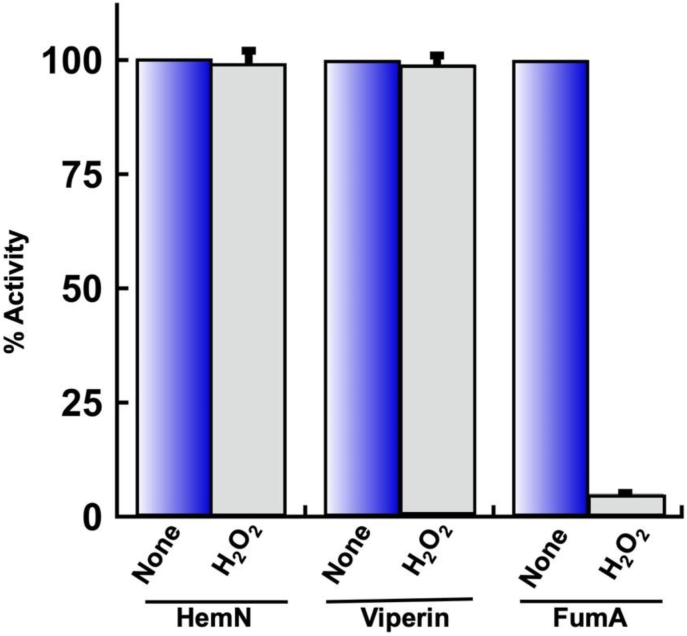
H_2_O_2_ does not inactivate HemN or viperin in vitro. HemN and viperin (30 μM) were assayed before and after 10 min treatment with 100 μM H_2_O_2_ in anoxic buffer. Fumarase A (100 nM) was inactivated by 1 μM H_2_O_2_ in 5 min(n = 3). Here and in subsequent figures the error bars represent the SEM of at least three independent experiments. See Fig. S1 for time courses of HemN and viperin exposures.

Purified HemN was directly exposed to H_2_O_2_ under anoxic conditions and then tested for SAM cleavage activity. The dithionite in the assay solution immediately eliminated residual H_2_O_2_. As little as 0.2 μM H_2_O_2_ is sufficient to activate OxyR and induce HemF inside cells [22]. However, in vitro even 100 μM H_2_O_2_ did not diminish HemN activity at all (Fig. 3 and Fig. S1). We verified that the H_2_O_2_ persisted throughout this exposure (Fig. S1). In contrast, the same H_2_O_2_ treatment immediately eliminated the activity of purified fumarase A, a [4Fe–4S] dehydratase [4]. We conclude that H_2_O_2_ does not disrupt the cluster of HemN and that OxyR control of *hemF* serves another purpose (Discussion).

A second RSE was examined. Viperin (virus inhibitory protein, endoplasmic reticulum-associated, interferon-inducible) is a human antiviral protein; it is also known as RSAD2 (radical S-adenosyl methionine domain containing 2) [26,[41], [42], [43]]. It can act upon CTP, converting it to an analogue that blocks viral replication, but the turnover number is low (0.2 min^−1^), leading some workers to propose that its authentic substrate may be something else [26,42,44,45]. Viperin homologs are present in many bacteria, archaea, and fungi. We selected viperin from *Methanofollis liminatans*, an obligately anaerobic archaeon that grows in wastewater bioreactors. This viperin has reliably exhibited activity in vitro [26], and because the source microbe is an anaerobe it seemed unlikely that it would possess special features to suppress ROS sensitivity. The viperin gene was cloned in a pETduet vector, co-expressed with the *isc* operon in catalase-deficient cells, and purified under anoxic conditions. The iron content indicated that 65% of the protein contained a [4Fe–4S] cluster (Table S2).

The purified enzyme generated increasing amounts of 5 dA with protein concentration and time (Fig. 2B). When incubated with 2 mM SAM, 50 mM DTT, and 5 mM dithionite for 10 min, 30 μM of enzyme yielded 60 μM 5 dA, indicating 2 turnovers per enzyme molecule. As was the case for HemN, viperin activity was unaffected by exposure to 100 μM H_2_O_2_ (Fig. 3 and Fig. S1). Again, assays confirmed that H_2_O_2_ persisted for the duration of the exposure time. Therefore, the in vitro data indicated that these RSEs, unlike dehydratases, are not damaged by H_2_O_2_. We estimate the difference in sensitivity to be > 100-fold.

### 3.2 RSE-dependent pathways remain unblocked in H_2_O_2_-stressed cells

*E. coli* Hpx^−^ mutants lack the catalase and peroxidases that normally degrade endogenous H_2_O_2_. When grown under aerobic conditions, these strains accumulate ∼ 1–2 μM H_2_O_2_ and fully induce the OxyR regulon [46]. Under these conditions the OxyR-dependent induction of HemF is required for efficient synthesis of catalase [22], and this observation had been the premise for the expectation that H_2_O_2_ might inactivate HemN. To test whether H_2_O_2_ poisons RSEs in general, we examined whether various RSE-dependent pathways remain functional in Hpx^−^ cells. To do so we tested whether these H_2_O_2_-stressed cells could continue to synthesize cofactors that derive from RSE-dependent pathways: heme (HemN) [33], biotin (BioB) [47], lipoic acid (LipA) [48], and thiamine (ThiC) [49].

**Fig. 4:**
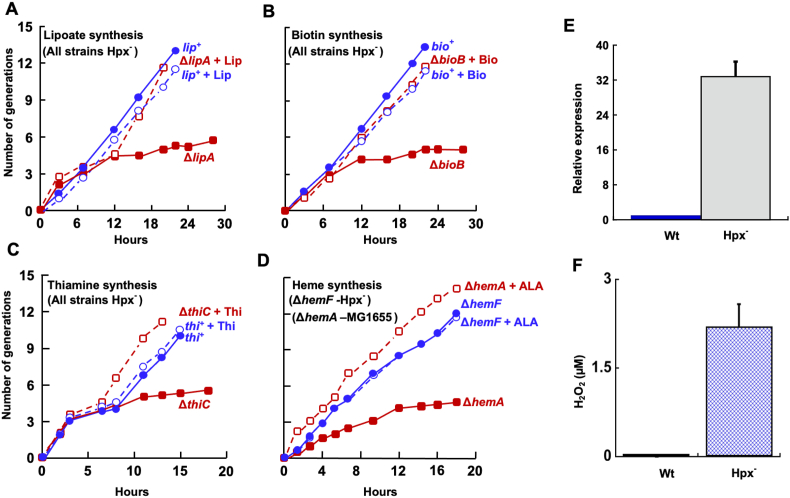
H_2_O_2_ does not poison radical SAM enzymes involved in cofactor biosynthesis in vivo. Hpx^−^ strains lack scavenging enzymes and experience stress from high levels of endogenous H_2_O_2_. Cells growing in glucose medium with vitamin supplements were washed and suspended in aerobic medium at time zero. The Hpx^−^ strains (blue lines) retained the ability to synthesize lipoic acid (A), biotin (B), thiamine (C), and heme (D), indicating that the RSEs LipA, BioB, ThiC, and HemF remained functional. Control Hpx^−^ strains with deletions of *lipA*, *bioB*, *thiC*, and *hemA* (red lines) show that defects in these pathways attenuate growth after four generations. Where indicated, cultures were supplemented with lipoate (+Lip), biotin (+Bio), thiamine (+Thi), or δ-aminolevulinate (+5-ALA). Eight amino acids were supplied to all media to compensate for H_2_O_2_-mediated damage to their biosynthetic pathways. See Fig. S2 for further quantitation (n = 3). Strains: SR026, SR028, SR030, SMA1489, SMA1140. (E) RT-PCR verified the presence of acute H_2_O_2_ stress. The OxyR-controlled gene *trxC* was induced 33-fold (n = 3). (F) H_2_O_2_ accumulation in cultures of WT and Hpx^−^ cells (n = 4). H_2_O_2_ equilibrates across the cytoplasmic membrane of Hpx^−^ strains, so that the extracellular level reflects the internal level, too. Strains: MG1655, LC106. (For interpretation of the references to colour in this figure legend, the reader is referred to the Web version of this article.)

Hpx^−^ cells were pre-cultured anaerobically into log phase in a minimal glucose medium, and they were then aerated. An Hpx-*hemF* strain was additionally used, to test the continued function of HemN. Aromatic amino acids were supplied, because in Hpx^−^ mutants H_2_O_2_ inactivates DAHP synthase, the first enzyme of the aromatic pathway [31]. Cofactors are present in excess in *E. coli*, so biosynthetic blocks manifest only after cells have been grown for multiple generations in the absence of vitamin supplements. We therefore cultivated mutants through repeated aerobic subcultures. Hpx^−^ strains that lacked *bioB, lipA*, or *thiC* and MG1655 lacking *hemA* stopped growing after approximately four generations, as expected. However, the growth of simple Hpx^−^ strains did not fail (Fig. 4A–D and Fig. S2). These data indicate that the RSEs HemN, BioB, LipA, and ThiC all retained enough activity inside H_2_O_2_-suffused cells that their pathways were fully functional. The Δ*hemA* strain was used as a control to verify that any block in heme synthesis causes an arrest in cell growth. (This strain can be kept alive under oxic conditions by δ-aminolevulinate supplementation, whereas strains blocked at the HemN/HemF step cannot be rescued by supplements.) The OxyR-controlled *trxC* gene [50] was induced 33-fold, confirming that the Hpx^−^ strains were experiencing severe H_2_O_2_ stress (Fig. 4E), and their culture media accumulated high levels of H_2_O_2_ (Fig. 4F). This in vivo result comports with the H_2_O_2_ resistance of HemN and viperin in vitro.

### 3.3 Molecular oxygen slowly inactivates viperin but not HemN in vitro

RSEs are known for their oxygen sensitivity, which precludes working with them in air-saturated buffers. Enzymes that are purified under oxic conditions lose their iron-sulfur clusters and are inactive. Nevertheless, these enzymes are functional inside fully aerobic cells, despite the fact that internal oxygen concentrations are as high as those outside the cell. To explore this apparent contradiction, we sought to quantify the oxygen sensitivity of HemN and viperin.

**Fig. 5:**
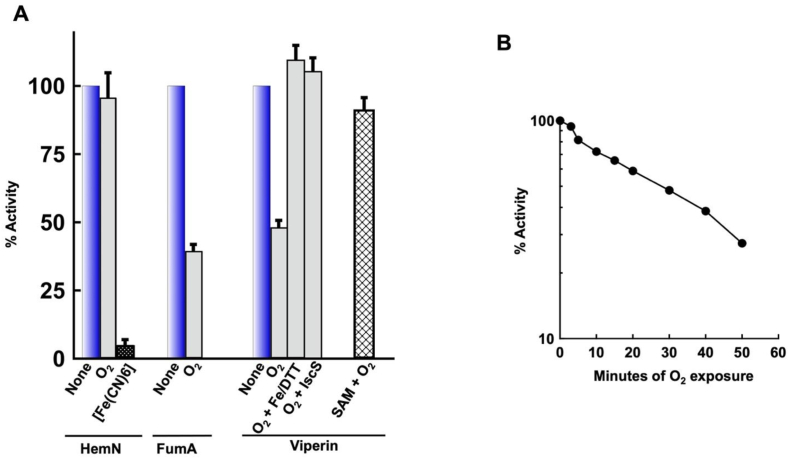
Damage to RSEs by molecular oxygen in vitro. (A) Enzymes were treated with O_2_ (200 μM; grey bars) at RT for 30 min in closed tubes inside the anaerobic chamber, prior to assay. HemN protein did not exhibit any activity loss (grey bars) compared to untreated enzyme (blue bars; see also Fig. S3). However, HemN was inactivated by a 10-min exposure to the potent oxidant ferricyanide (100 μM) (black bar). In contrast, oxygen gradually inactivated both viperin and fumarase A (n = 3). Damaged viperin was fully reactivated by subsequent treatment with 0.5 mM ferrous ammonium sulfate and 5 mM DTT; incubation with IscS was without further benefit, indicating that the damaged clusters were in the [3Fe–4S]^**+**^ state. Hatched bars: The presence of 2 mM SAM during O_2_ exposure blocked viperin damage. (B) Time course of viperin damage by O_2_. (For interpretation of the references to colour in this figure legend, the reader is referred to the Web version of this article.)

HemN was exposed to 200 μM O_2_ in buffer and then assayed. Dithionite in the assay mix rapidly reduces oxygen, so that O_2_ was not present during the assay itself. Surprisingly, HemN did not lose significant activity within the 20 min of oxygen exposure (Fig. 5A and Fig. S3A). In contrast, the dehydratase fumarase A lost about half of its activity during that period, consistent with previous reports [27].

Molecular oxygen can be reduced by outer-sphere electron transfer, so we were skeptical that the HemN cluster could be fully protected by physical shielding. Indeed, when ferricyanide was added, the enzyme lost virtually all activity (Fig. 5A). Ferricyanide is a much more potent univalent oxidant (Eo’ = + 0.42 V [51]) than is molecular oxygen (Eo’ = - 0.16 V) [52,53], and we infer that an energetic barrier contributes to the oxygen resistance of the HemN cluster.

In contrast, the viperin cluster did lose activity during oxygen exposure (Fig. 5A). Neither catalase nor superoxide dismutase was protective (Fig. S3B), confirming that O_2_ itself was responsible. The half-time of inactivation was approximately 28 min (Fig. 5B), which is comparable to that of fumarase A (t_1/2_ = 20 min) (Fig. 5A). Interestingly, the inactivated enzyme could be restored to full activity by treatment with ferrous iron and DTT. This indicates that the cluster had disintegrated to a [3Fe–4S] form but no further, as sulfur atoms did not need to be provided to reassemble a functional cluster. The same is true of oxidized fumarase, whether molecular oxygen, superoxide, or H_2_O_2_ is the oxidant. (Attempts to visualize the oxidized cluster by EPR presented complex data due to the incomplete occupancy of the original enzyme pool.)

Substrates can protect dehydratase clusters from oxidation [4,54]. We observed that the addition of SAM had a substantial stabilizing effect upon the viperin cluster in the presence of oxygen (Fig. 5A). SAM binds RSE clusters in bidentate fashion. Several mechanisms of protection could be at work: SAM could keep oxygen at a distance, it could alter the reduction potential of the cluster, or it could simply hold the oxidized cluster together until dithionite becomes available to re-reduce it in the assay cocktail. Regardless, the stabilization by SAM likely contributes to RSE stability in vivo, too.

### 3.4 Viperin and HemN are substantially resistant to superoxide

The superoxide concentration inside unstressed *E. coli* (0.2 nM) is estimated to be much lower than that of H_2_O_2_ (50 nM), but the rate constant with which it reacts with dehydratase clusters can be 1000-fold higher [53]. When anaerobes are aerated, it is superoxide rather than H_2_O_2_ or O_2_ per se that inactivates this enzyme family. We tested whether superoxide also inactivates RSE.

**Fig. 6:**
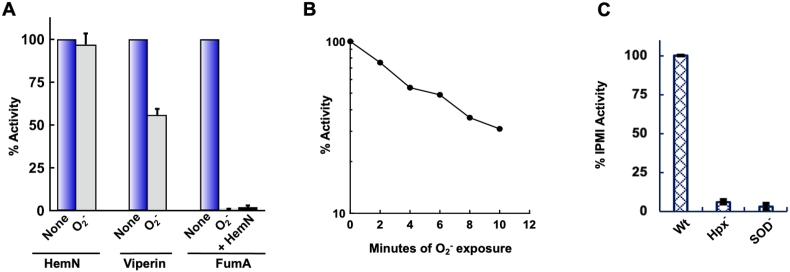
Superoxide is not a potent oxidant of HemN or viperin in vitro. (A) HemN and viperin (30 μM) were exposed to superoxide generated by xanthine oxidase for 6 min, prior to assay. Fumarase was exposed for 1 min. The addition of HemN did not block the inactivation of fumarase, verifying that the HemN preparation did not possess superoxide-scavenging activity (n = 3). (B) Time course of viperin damage by O_2_^−^. Approximately 20% of the inactivation may be due to direct damage by O_2_ (compare to Fig. 5B). See Fig. S4 for time course of HemN damage. (C) Enzyme activity of [Fe–S]-cluster dependent IPMI in Hpx^−^ and SOD^−^ cells (n = 3). The low activity creates a biosynthetic deficit (Fig. S4B). Strains: MG1655, LC106, KI232.

Xanthine oxidase was used as a continuous source of superoxide [5]. HemN was unaffected (Fig. 6 and Fig. S4), but viperin gradually lost activity, with a half-time of 6.6 min. The inclusion of SOD suppressed the rate of inactivation to that seen with O_2_ alone. Strikingly, the rate at which superoxide inactivated viperin did not approach that of fumarase A, which was fully inactive (<2% starting activity) within 1 min. To be certain that the RSEs did not copurify with any SOD activity, we showed that the addition of the HemN preparation did not slow the inactivation of fumarase. We estimate that viperin is > 40-fold more resistant to O_2_^−^ than is fumarase, while HemN is > 500-fold more resistant.

**Fig. 7:**
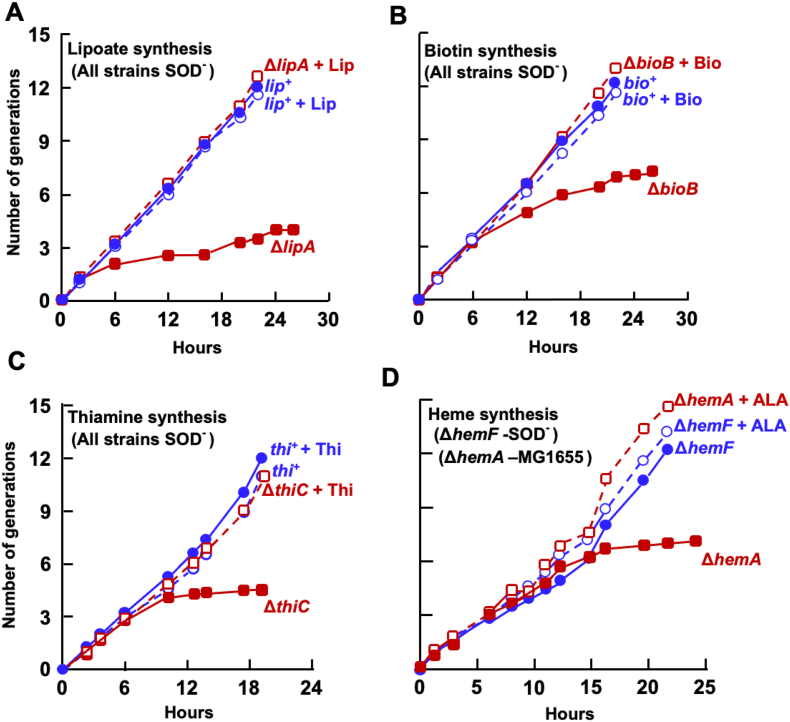
Superoxide does not poison radical SAM enzymes involved in cofactor biosynthesis in vivo. Superoxide dismutase-deficient (SOD^−^) strains cultured in aerobic glucose medium with vitamin supplements were washed and suspended in glucose medium at time zero. The SOD^−^ strains (blue lines) retained the ability to synthesize lipoic acid (A), biotin (B), thiamine (C), and heme (D), indicating that the RSEs LipA, BioB, ThiC, and HemN remained functional. Control SOD^−^ strains with deletions of *lipA*, *bioB*, *thiC*, and *hemA* (red lines) show that defects in these pathways would have attenuated growth after four generations. The *hemF* mutation makes heme synthesis fully dependent upon HemN. Where indicated, cultures were supplemented with lipoate (+Lip), biotin (+Bio), thiamine (+Thi), or δ-aminolevulinate (+5-ALA). Eight amino acids were provided to compensate for superoxide-mediated damage to dehydratases in biosynthetic pathways. See Fig. S5 for further quantitation (n = 3). Strains: SR032, SR034, SR036, SR038, KI232, SMA1140. (For interpretation of the references to colour in this figure legend, the reader is referred to the Web version of this article.)

The sensitivity of RSEs to superoxide was also tested in vivo. Mutants of *E. coli* that lack cytoplasmic SOD exhibit several phenotypes that arise from the inactivity of dehydratases [30] They are auxotrophs for branched-chain amino acids because superoxide damages dihydroxyacid dehydratase [6] and isopropylmalate isomerase (IPMI), and they cannot catabolize TCA-cycle substrates such as acetate because superoxide inactivates aconitase [54] and fumarase [55]. These effects were confirmed by IPMI activity measurements in the experiments here (Fig. 6C), as well as the inability of SOD^−^ mutants to grow without branched-chain amino acid supplements (Fig. S4B). We found that these SOD^−^ mutants, however, could grow indefinitely in defined media that lacked thiamine, biotin, lipoate, or (in a *hemF* background) heme (Fig. 7 and Fig. S5). Control strains that lacked *bioB*, *lipA*, *thiC*, and *hemA* stopped growing after several generations. Therefore, these enzymes remain functional in an SOD^−^ mutant—which accords with RSE resistance to superoxide in vitro.

### 3.5 RSE sensitivity to soft metals

The exposed situation of their [4Fe–4S] clusters also sensitizes dehydratases to certain non-oxidizing small molecules. This effect is of special interest because phagocytes dowse captured bacteria not only with ROS but also with copper and nitric oxide.

Dehydratase clusters were originally identified as primary biological targets of copper(I) because copper-stressed cells were unable to synthesize branched-chain amino acids or to grow on non-fermentable carbon sources [18]. In vitro studies showed that copper progressively displaced iron from the exposed clusters, inactivating the enzyme; activity was restored when the clusters were reconstituted. This behavior was ascribed to the thiophilic nature of copper, and indeed other soft metals replicated the effect, in vitro and in vivo [18]. More recently, Azzouzi et al. and Djoko and McEwan observed that the photosynthetic bacterium *Rubrivivax gelatinosus* and *Neisseria gonorrhea* both excrete porphyrins when they are toxified by copper [[19], [20], [21]]. Citing the copper sensitivity of dehydratase clusters, the authors suggested that copper might also poison HemN, although no enzymatic studies were performed.

**Fig. 8:**
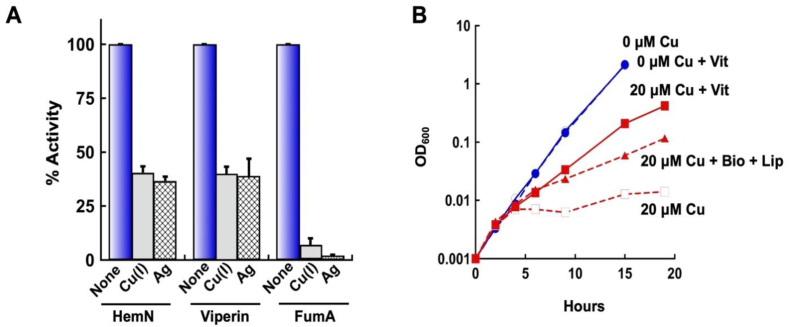
Copper inactivates RSEs in vitro and in vivo. (A) Purified HemN, viperin, and fumarase A were exposed to 100 μM of Cu(I) or Ag(I) for 5 min anoxically at RT and then assayed for SAM-cleavage activity (n = 3). See Fig. S6 for time courses. (B) The LEM33 strain lacking copper-efflux systems (*ΔcopA ΔcueO Δcus*) was cultured in glucose medium to which copper (20 μM, red curves) was added at time zero. Lipoate, biotin, and thiamine were supplied as indicated (+Vit). The medium contained branched-chain amino acids to compensate for copper-mediated damage to that biosynthetic pathway. See Fig. S6 for further quantitation (n = 3). (For interpretation of the references to colour in this figure legend, the reader is referred to the Web version of this article.)

To test this idea, we incubated HemN anoxically with 100 μM Cu(I). The enzyme lost half its activity in 5 min (Fig. 8A and Figs. S6A–B). Viperin was inactivated at the same rate. The same dose inactivated 90% of fumarase A. Because the experiment was conducted in the presence of ascorbate and in the absence of oxygen, copper was maintained in its reduced valence, ensuring that the damage did not depend upon the oxidation of the cluster. Further, silver(I) is a redox-inactive, thiophilic analog of copper(I). It, too, was able to inactivate viperin, albeit somewhat more slowly than it did fumarase A (Fig. 8A).

Like most bacteria, *E. coli* does not employ copper in any cytoplasmic enzymes, and it possesses efflux pumps to keep copper out of this compartment [56]. The toxicity of Cu(I) was examined in *copA cus cueO* mutants of *E. coli* that lack these systems. The mutants were cultured in anoxic glucose medium supplemented with the branched-chain amino acids. The addition of copper blocked growth within approximately three generations (Fig. 8B and Fig. S6C). The addition of lipoate, biotin, and thiamine greatly improved the growth of the copper-treated cells, while having no impact upon the growth rate of untreated cells. We infer that copper impaired these biosynthetic pathways. When the vitamins were individually tested, it appeared that thiamine supplements were the most critical for growth.

### 3.6 Nitric oxide can inhibit RSEs

**Fig. 9:**
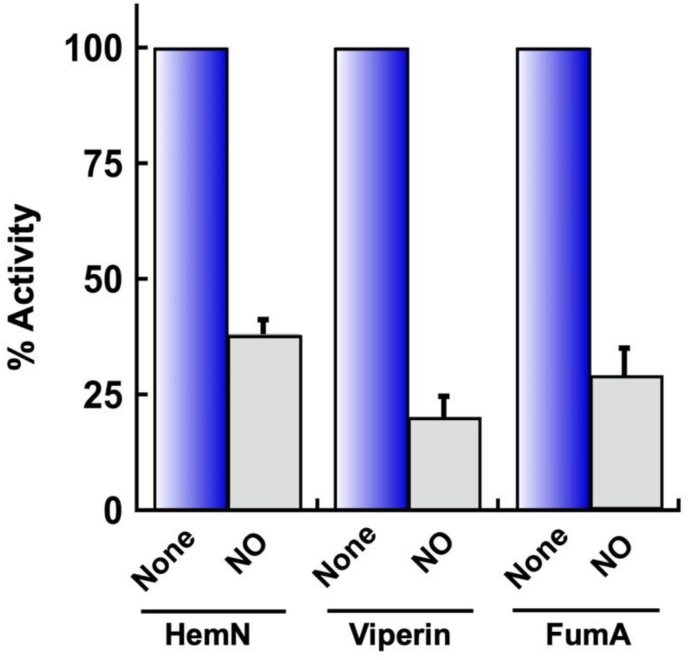
HemN and viperin can be inactivated by nitric oxide (NO). Purified HemN and viperin (30 μM) and fumarase A (100 nM) were exposed to NO anoxically at RT for 10 min (n = 3). Activity was then measured. The NO was generated by 100 μM of DEA-NONOate. See Fig. S7 for time courses.

Nitric oxide (NO) forms complexes with iron atoms. The nitrosylation of [4Fe–4S] clusters has been detected in studies of dihydroxyacid dehydratase [57], aconitase [58], and endonuclease III [59]. To our knowledge NO reactivity with RSEs has not been tested. We exposed purified enzymes to NO that was released by the NO donor DEA-NONOate at room temperature. NO inactivated viperin, HemN, and fumarase at similar rates (Fig. 9 and Fig. S7).

Primary targets of NO in *E. coli* include the cytochrome oxidases that terminate the respiratory chain [60,61]; ribonucleotide reductase is also affected by low doses [62]. Because those actions block growth, we were unable to perform growth tests that might specifically detect the effects of NO upon RSEs in vivo.

## 4 Discussion

**Fig. 10:**
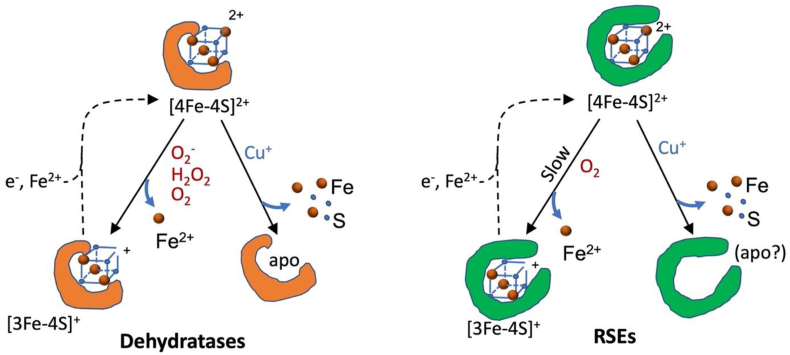
Radical SAM enzymes differ from iron-sulfur dehydratases in their sensitivity to oxidants. (Left) Dehydratase clusters are oxidized slowly by oxygen and far more rapidly by superoxide and hydrogen peroxide. A single iron atom is lost; reactivation requires reduction and remetallation. Soft metals displace all four iron atoms, and reactivation requires de novo cluster synthesis. (Right) The present study indicates that RSE clusters can also be oxidized by molecular oxygen but are resistant to superoxide and hydrogen peroxide. The basis of resistance is unclear (see text). RSEs remain vulnerable to soft metals.

Our results indicate that superoxide and hydrogen peroxide do not attack RSE clusters, that oxygen does so at a pace that is too modest to disrupt RSE function in vivo, and that copper and possibly nitric oxide pose more of a threat (Fig. 10). Our in vitro experiments employed only two enzymes, but the in vivo analysis included three more.

### 4.1 Why does *E. coli* induce HemF during H_2_O_2_ stress?

The impetus for this study was our observation that *hemF* is a member of the *E. coli* OxyR regulon [22]. In that work we suggested two possible explanations: that H_2_O_2_ impairs either the stability or the assembly of HemN clusters. We now suspect that during periods of H_2_O_2_ stress iron may become too scarce to ensure HemN cluster assembly.

When HemN and HemF were first determined to be isozymes, the oxygen sensitivity of purified HemN prompted workers to presume that it was used exclusively in anoxic habitats. This statement is still found in literature (EcoCyc). However, ribosomal sequencing implied that in unstressed aerobic cells, the primary coproporphyrinogen III oxidase is actually HemN, as it is synthesized at a rate four-fold higher than HemF [63]. Accordingly, we observed that a *hemF* mutant exhibits no defect in the activation of heme-dependent enzymes [22].

Instead, HemF usurps the role of HemN specifically when the cell experiences H_2_O_2_ stress. In that circumstance, the OxyR response induces catalase approximately 30-fold. Because basal-level catalase initially comprises about 10% of the cellular heme content, during H_2_O_2_ stress the total cellular demand for heme synthesis may rise three-fold (calculated from Refs. [22,63]). This situation might justify induction of coproporphyrinogen III oxidase activity—but further synthesis of HemN might become problematic, because the cell simultaneously strives to diminish its pool of loose iron. OxyR induces Dps, a mini-ferritin, that sequesters iron and thereby suppresses Fenton-mediated DNA damage [[64], [65], [66]]. That adjustment is necessary to keep the cell alive. However, it makes it difficult for the cell to provide iron-based cofactors to nascent proteins. Indeed, experiments show that if the OxyR regulon is genetically activated, even in the absence of H_2_O_2_ stress, catalase activation becomes inadequate—unless HemF is also induced [22].

The H_2_O_2_-stressed cell invokes several strategies to accommodate the decline in iron pools: OxyR induces ferrochelatase, to insert scarce iron into porphyrins [22]; the Suf system, which is more efficient than the Isc system at assembling clusters when iron is limited [35,67]; and the MntH manganese importer, which allows Mn(II) to substitute for Fe(II) in mononuclear enzymes [68,69]. The ClpSAP protease is induced to keep Dps from draining absolutely all the iron inside the cell [28]. We suspect that the emergency induction of HemF is a similar adjustment, to ensure that iron sequestration does not stand in the way of the critical induction of catalase activity. The shift toward a reliance on manganese rather than iron for the oxidase activity exploits the simultaneous induction of the manganese importer.

### 4.2 Why don't O_2_^−^ and H_2_O_2_ damage the clusters of RSEs?

Our expectation that HemN would be ROS-sensitive was also based on the ROS sensitivity of iron-sulfur dehydratases, which like RSEs have exposed clusters. We do not yet have a definitive answer as to why RSEs turn out to be ROS-resistant. Several factors may be involved. The cluster potential for the +2-to-+3 transition that leads to RSE inactivation is not known, and it will be difficult to determine as the oxidized cluster is unstable. However, the fact that the viperin cluster can be oxidized by molecular oxygen argues that H_2_O_2_ should be a sufficient thermodynamic oxidant, too. It seems more likely that some aspect of the physical disposition of the cluster interferes with ROS oxidation. Cluster oxidation by superoxide and H_2_O_2_, but not by O_2_, requires the direct binding of the oxidant to iron [53], and so shielding by polypeptide or by small molecules is protective [4,54,70]. The bridging and/or liganding sulfur atoms of the cluster seem sufficiently exposed that copper can attack them, but the catalytic iron atom might still be occluded. The availability of local proton-donating and/or -accepting residues could also play a role: Superoxide is anionic at physiological pH but must be protonated in order to abstract an electron from a cluster, while peroxide must be deprotonated in order to bind it [71]. The RSE crystal structures that are in hand do not satisfactorily answer this question.

### 4.3 Why do oxygen-sensitive RSEs function inside oxic cells?

RSEs are famously oxygen sensitive. Oxic conditions cannot be used to purify or handle active enzyme; after oxygen exposure, clusters must be reassembled to restore activity. The analysis above indicates that molecular oxygen itself, rather than ROS derived from it, damages the cluster.

This cluster damage is distinct from the oxygen adduction that can poison a different class of radical-based enzymes. Pyruvate:formate lyase, for example, features a solvent-exposed glycyl radical which oxygen rapidly adducts, leading to polypeptide cleavage [72]. This inactivating injury occurs both in vitro and in vivo and precludes the use of such enzymes by aerobic organisms. Instead, RSEs are more similar to B_12_-type enzymes: Both families generate adenosyl radicals only as momentary catalytic intermediates, and they do so inside occluded active sites that are likely to exclude oxygen [1,[73], [74], [75], [76], [77], [78]].

Therefore, the oxygen sensitivity of RSEs is observed even when the enzyme is not actively processing substrate. In this study we quantified the sensitivities of the resting enzymes. Oxygen inactivated [4Fe–4S]^2+^ viperin at a rate comparable to [4Fe–4S]^2+^ dehydratases; in both cases, reactivation is achieved by Fe(II)/DTT treatment, indicating that oxidation converted each of them to a [3Fe–4S]^+^ form. Dehydratases remain functional in oxic cells, as their high steady-state activity represents the balance between their relatively slow oxidation (t_½_ ∼ 20 min) and quick intracellular reactivation (t_½_ ∼ 5 min) [7,8,27,53]. We expect the same to be true of RSEs. Other features may also protect RSEs in vivo. First, viperin sensitivity to oxygen was suppressed by the binding of SAM, which is at saturating levels inside bacteria. Second, it is possible that the reduction of the cluster to its functional [4Fe–4S]^+^ state by flavodoxins would minimize the time spent in the vulnerable [4Fe–4S]^2+^ form. Finally, cofactor biosynthetic pathways are resilient. Because a cofactor biosynthetic bottleneck does not impair growth for several generations, the cell has the opportunity to induce the RSE and relieve the deficit before growth stops. This situation contrasts with that for amino acid biosynthesis, where a bottleneck impairs growth immediately [30] and can block any compensatory induction of the pathway enzymes. Collectively, these features of RSE enzymes apparently enable their continued function in oxic habitats.

Interestingly, workers have proposed that the RSEs that activate PFL and the anaerobic ribonucleotide reductase may be quite oxygen sensitive in vivo [79]. If so, their inactivation by oxygen would comprise a control mechanism that avoids the fruitless activation of PFL and RNR inside aerobic cells. This scheme would require that these RSEs be much more sensitive than the RSEs that we sampled. We hope that the experimental strategies that we used might enable other workers to similarly quantify the sensitivities of other RSEs.

### 4.4 RSEs are among the targets of copper

Many bacteria possess inducible copper-efflux pumps, implying that copper is a threat in many microbial habitats [56,80]. Our data indicate that RSE enzymes join [4Fe–4S] dehydratases as the targets that these systems evolved to protect. Previous work in other labs detected the excretion of porphyrins when cells were exposed to toxic levels of copper [[19], [20], [21]]. It was inferred that copper might be able to inactivate HemN, and we have now confirmed this directly. The in vitro experiments with viperin, and the growth studies involving other vitamins, indicate that copper likely poisons other RSEs as well. Copper-rich habitats may include the phagosome, into which copper is pumped in an effort to suppress the growth of captive bacteria [[81], [82], [83], [84]]. Nitric oxide is also generated there [85,86], and our enzyme data suggests that RSEs may be among its targets, too. It would be of interest to test more directly whether RSE-dependent pathways are compromised in phagocytosed bacteria.

In sum, this study suggests that the solvent exposure of RSE clusters makes them vulnerable to a number of small molecules—soft metals, NO, and oxygen—but not to superoxide and H_2_O_2_ (Fig. 10). The known targets of the latter species are still iron-sulfur dehydratases, mononuclear Fe(II) enzymes, and DNA [10].

## Declaration of competing interest

The authors declare no potential conflict of interest.

## Data Availability

No data was used for the research described in the article.
