# Supplementary material for: The vulnerability of radical SAM enzymes to oxidants and soft metals
Source: Redox Biol. 2022 Oct 7;57:102495. doi: 10.1016/j.redox.2022.102495 (PMC9576991; doi:10.1016/j.redox.2022.102495)
Supplement: Multimedia component 1 [file mmc1.docx]

**Supplementary data**

**The vulnerability of radical SAM enzymes to oxidants and soft metals.**

Sanjay Kumar Rohaun and James A. Imlay#

Department of Microbiology, University of Illinois, Urbana, IL 61801

#Corresponding author: (217)-333-5812; fax 217-244-6697; jimlay@illinois.edu

Running title: Oxidative damage to radical SAM enzymes

Keywords: reactive oxygen species, copper, nitric oxide, iron-sulfur clusters


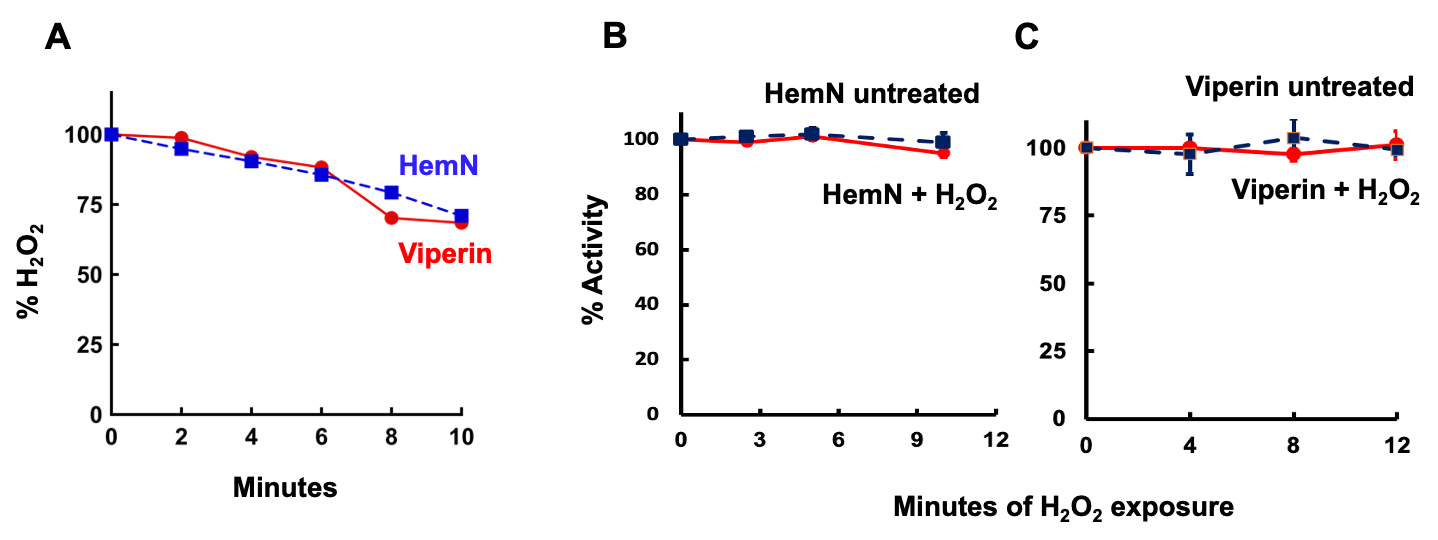


**Fig. S1. H_2_O_2_ is stable in the enzyme-peroxide mix.** The Amplex Red/horseradish peroxidase assay was used to quantify hydrogen peroxide in the HemN (squares) and viperin (circles) enzyme-peroxide incubations. (B-C) Time course of RSE damage by H_2_O_2_. (A) HemN and (B) viperin (30 μM) were assayed after treatment with 100 μM H_2_O_2_ (red line with circles) or anoxic buffer (dotted line with squares) for different time at RT anaerobically (n = 3).


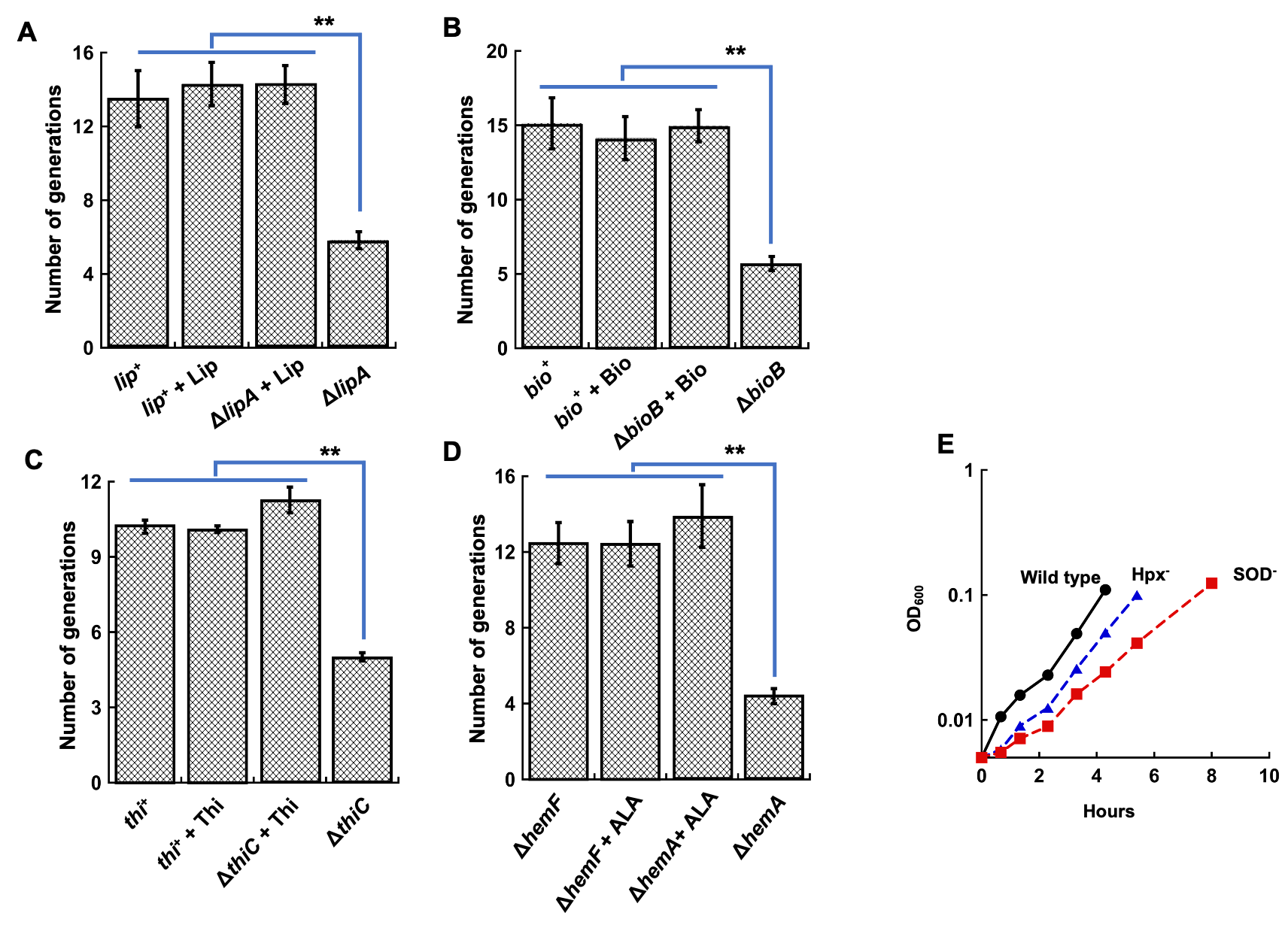


**Fig. S2. Endogenous levels of H_2_O_2_ do not inactivate RSE.** All cells used are Hpx^-^ except *hemA*. During H_2_O_2_ stress as in Fig. 4, cell growth was assessed to test for effects on RSEs involved in cofactor synthesis (as labeled). (A-D) Cell growth (as number of generations) with standard error (from 3 independent experiments) are shown for 20 hr (*lipA* and *bioB*) or 15 hr (*thiC*, *hemA*, and *hemN*) time points, analysed by one way ANOVA test, p < 0.01. Strains: SR026, SR028, SR030, SMA1489, SMA1140. (E) Sample growth curves showing differences among WT (MG1655, solid line with circles), Hpx^-^ (LC106, dotted line with triangles), and SOD^-^ (KI232, dotted line with squares) strains in oxic minimal glucose media with 8 amino acids. Doubling times with SEM are 0.96 +/- 0.04 hrs for WT, 1.4 +/- 0.13 hrs for Hpx^-^, and 1.7 +/- 0.07 hrs for SOD^-^.


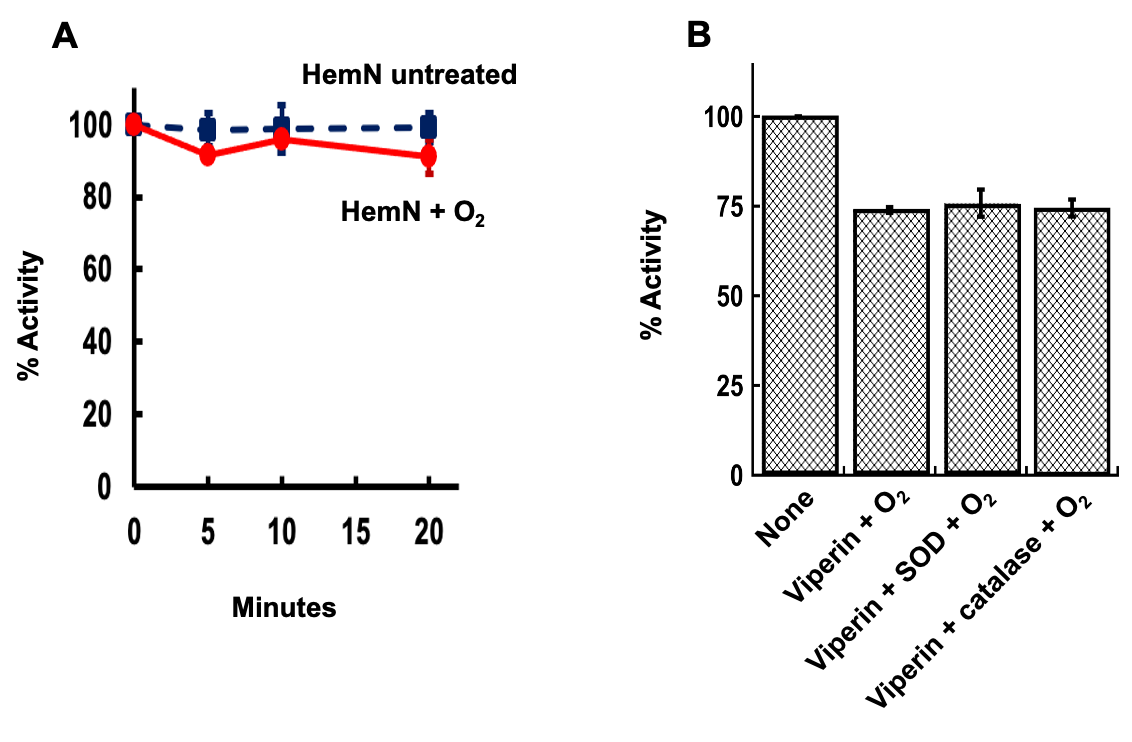


**Fig. S3. Effect of oxygen on RSE.** (A) Time course for effect of O_2_ on HemN activity. HemN (30 μM) enzyme was treated with 200 μM O_2_ (solid line with circles) or anoxic buffer (dotted line with squares) at RT, prior to assay in closed tubes inside the anaerobic chamber. (B) SAM cleavage activity of viperin under different treatments. Where indicated, superoxide dismutase (SOD, 500 U/ml) or catalase (500 U/ml) were added to viperin prior to treating with O_2_ (200 μM for 10 min at RT). SOD and catalase were not able to protect the enzyme. n = 3 for all the experiments.


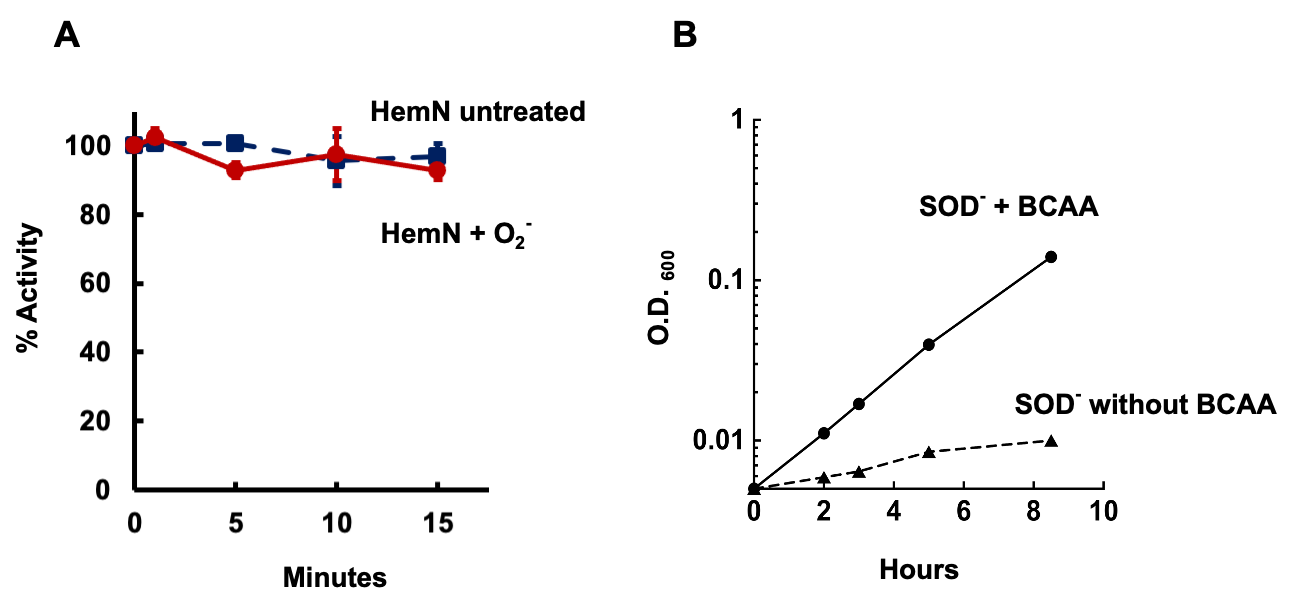


**Fig. S4. Superoxide effect on HemN enzyme.** (A) HemN (30 μM) was exposed to superoxide generated by xanthine oxidase (dotted line with squares) or anoxic buffer (solid line with circles) for different time, prior to assay (n = 3). 5dA formation activity is plotted after treatment with O_2_^-^. (B) Growth of SOD mutant (KI232) with or without BCAA (branch chain amino acid supplement), showing BCAA synthesis pathway block under superoxide stress.


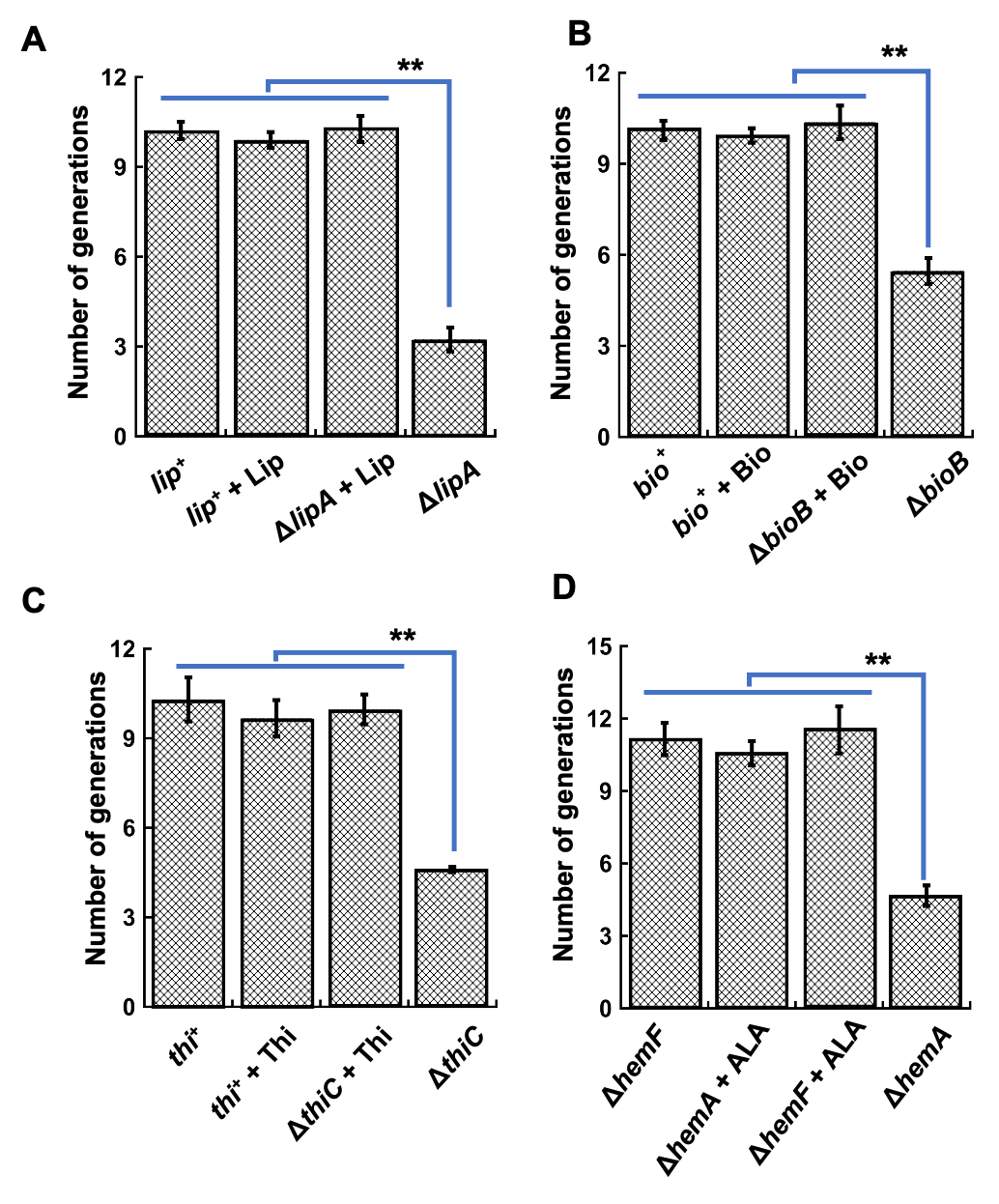


**Fig. S5. Superoxide effect on RSE in vivo.** All cells used are SOD^-^ except *hemA*. Aerobic growth curves were conducted during superoxide stress (same as in Fig. 7) to test for effects on different RSEs (LipA, BioB, ThiC, and HemN; A-D). Control experiments with *lipA*, *bioB*, *thiC*, and *hemA* mutants demonstrate that these pathways are essential for growth without supplements. (As shown in Fig. 7, residual cofactors inside the cell allow these mutants to grow for several generations before growth ceases.) The *hemF* mutation makes heme synthesis fully dependent upon HemN. The number of generations achieved by the 20-hr time point were plotted. Standard error was calculated from 3 independent experiments and statistical significance was calculated using one way ANOVA test, p < 0.01. Strains: SR032, SR034, SR036, SR038, KI232, SMA1140.


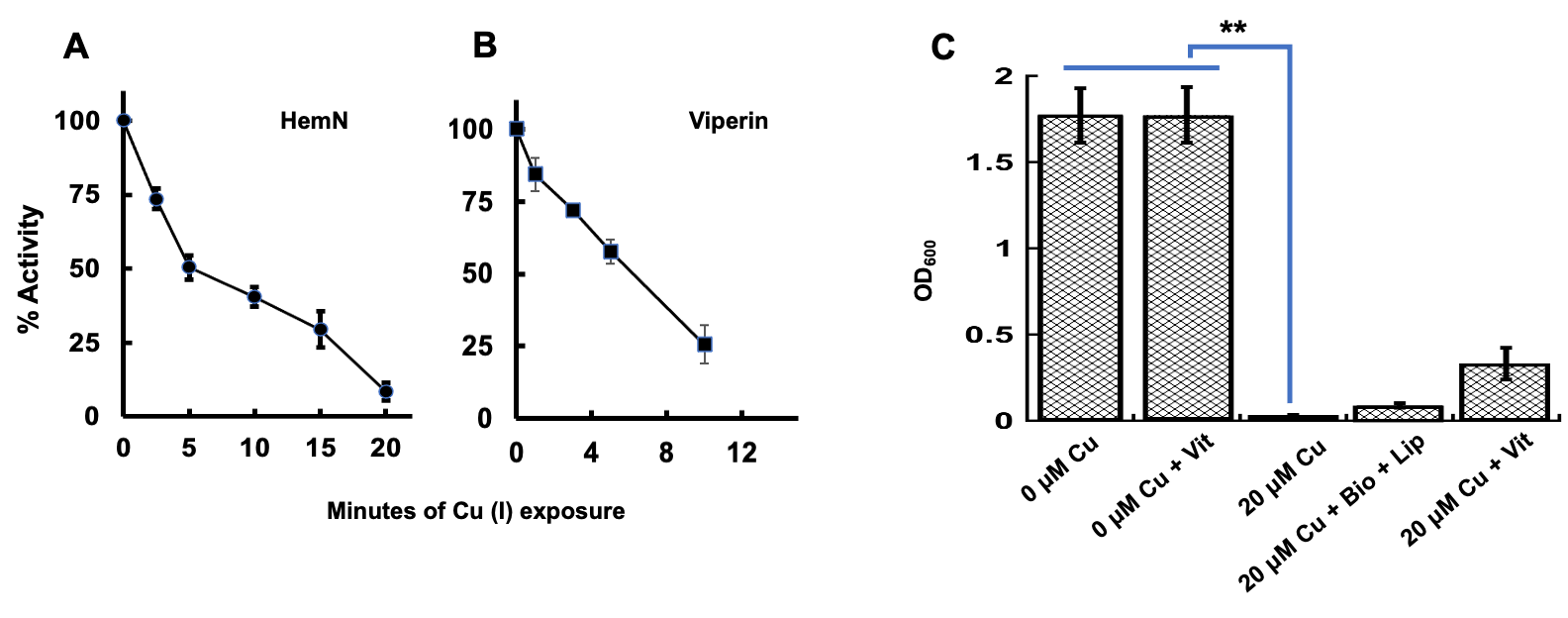


**Fig. S6. Effect of Cu(I) on RSE.** (A-B) Purified HemN (A), and viperin (B) were exposed to 100 μM Cu(I) anoxically at RT and then assayed for SAM-cleavage activity (n = 3). (C) The LEM33 strain lacking copper-efflux systems (*∆copA ∆cueO ∆cus*) was cultured in glucose medium with branched-chain amino acids. Growth was monitored at 600 nm ± copper (20 μM) and ± vit [lipoate (Lip), biotin (Bio), and thiamine]. Error bars and statistical significance (one way ANOVA test, p < 0.01) are shown for the 15 hr time point from three independent experiments.


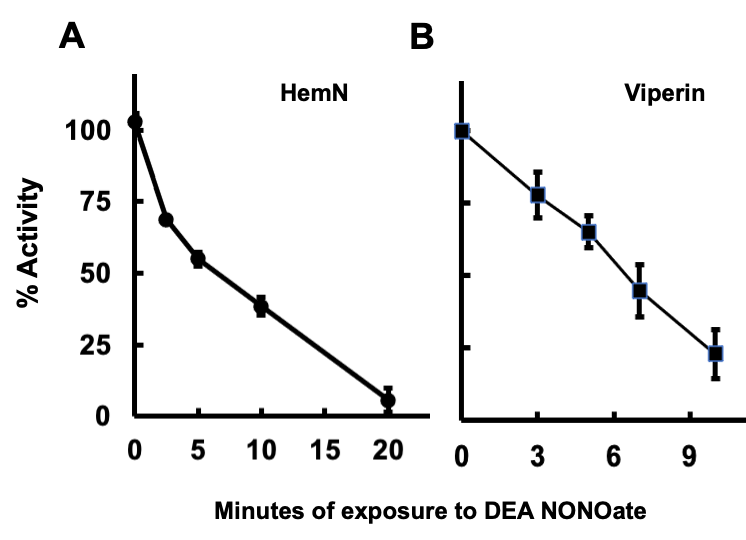


**Fig. S7. Nitric oxide (NO) inhibition of RSE.** (A) Purified HemN and (B) viperin (30 μM) were exposed to NO anoxically at RT for varying time points (n = 3). Activity was then measured. The NO was generated by 100 μM of DEA-NONOate.

31 **Table S1. Radical SAM enzymes present in *E. coli* (source: https://biocyc.org).**

32

| **Gene** | **Enzyme** | **Pathway/process (if known)** |
| --- | --- | --- |
| *aslB* | putative anaerobic sulfatase maturation enzyme | sulfatase maturation |
| *bioB* | biotin synthase | biotin synthesis |
| *epmB* | lysine 2,3-aminomutase | EF-P modification |
| *hemN* | coproporphyrinogen III dehydrogenase | heme synthesis |
| *hemW* | heme chaperone HemW | insertion of heme into hemoprotein |
| *lipA* | lipoyl synthase | lipoate synthesis |
| *miaB* | isopentenyl-adenosine A37 tRNA methylthiolase | tRNA modification |
| *moaA* | GTP 3',8'-cyclase | molybdopterin biosynthesis |
| *nrdG* | ribonucleoside-triphosphate reductase activating protein | anaerobic ribonucleoside triphosphate reduction |
| *pflA* | pyruvate formate-lyase activating enzyme | activation of pyruvate formate lyase |
| *pflC* | putative glycyl radical activating enzyme | - |
| *phnJ* | carbon-phosphorus lyase | methylphosphonate degradation |
| *queE* | putative 7-carboxy-7-deazaguanine synthase | queuosine synthesis |
| *rimO* | ribosomal protein S12 methylthiotransferase | ribosomal protein modification |
| *rlmN* | rRNA/tRNA methyltransferase | rRNA and tRNA modification |
| *thiC* | phosphomethylpyrimidine synthase | thiamine diphosphate synthesis |
| *thiH* | 2-iminoacetate synthase | thiamine diphosphate synthesis |
| *ybiY* | putative glycyl-radical activating enzyme | - |
| *ydeM* | putative anaerobic sulfatase maturation enzyme | - |
| *ygiQ* | unknown function | - |
| *yhcC* | unknown function | - |
| *yjjW* | putative glycyl-radical enzyme activating enzyme | - |

33

34

35

36

**Table S2. Iron content of purified recombinant RSE.** Ferene-S assay was used to measure iron content in the purified protein after overexpression in SR021 strain.

| **Protein** | **Calculated iron (µM)** | **Theoretical iron (µM)** | **% occupancy** |
| --- | --- | --- | --- |
| HemN (5 µM) | 11.05 ± 0.30 | 20 | 55 % |
| Viperin (5 µM) | 12.90 ± 0.26 | 20 | 65 % |
